# Catalytically active inclusion bodies of *Bacillus clausii* laccase protein recombinantly produced in *E. coli* for dye decolorization

**DOI:** 10.1186/s12934-026-02949-4

**Published:** 2026-03-06

**Authors:** Daniel Romero-Martínez, Francisco Gasteazoro, Luis F. Cofas-Vargas, Enrique García‑Hernández, Oscar González-Davis, Guadalupe Zavala, Diego Rosiles-Becerril, Rafael Vazquez-Duhalt, Mauricio A. Trujillo‑Roldán, Norma A. Valdez‑Cruz

**Affiliations:** 1 Departamento de Bionanotecnología, Centro de Nanociencias y Nanotecnología, Universidad Nacional Autónoma de México, Carr. Tijuana-Ensenada km 107, 22860 Ensenada, BC México; 2 Departamento de Biología Molecular y Biotecnología, Instituto de Investigaciones Biomédicas, Universidad Nacional Autónoma de México, Ensenada, México; 3 Instituto de Química, Universidad Nacional Autónoma de México, Ciudad Universitaria, Ciudad de México, México; 4 Institute of Fundamental Technological Research, Polish Academy of Sciences, Warsaw, Poland; 5 Unidad de Microscopía, Instituto de Biotecnología, Universidad Nacional Autónoma de México, Cuernavaca, Morelos México

**Keywords:** Laccase, Auto-immobilized, Nanoparticles, Contaminants, Dyes

## Abstract

**Introduction:**

Pollution causes disease and premature death globally. Various strategies, including biological methods like enzymes, are used to treat pollutants. The production of recombinant proteins in *E. coli* often results in their aggregation as inclusion bodies (IBs), enriched with the protein of interest. We developed a novel strategy for producing an immobilized bacterial laccase through self-assembly into catalytically active IBs (CatIBs) in *E. coli*. Our target was the outer spore-coat protein CotA from *Bacillus clausii*, a halotolerant and pH-stable laccase suitable for scalable applications in wastewater treatment. Two constructs were designed: Lac-17 (without fusion tags) and Lac-40EL (fused to a synthetic 35-amino-acid peptide, named as 40EL), both expressed in *E. coli* BL21(DE3).

**Results:**

Both strains showed similar growth patterns, though Lac-17 yielded more total recombinant protein (~ 163 mg/L vs. ~127 mg/L). However, Lac-40EL formed CatIBs with up to 22-fold higher enzymatic activity than Lac-17, despite ~ 85% of the recombinant protein being in the insoluble fraction in both cases. Structural models suggest that the 40EL peptide forms an exposed double α-helix that promotes ordered aggregation without hindering the active site of the enzyme. Unlike the soluble fractions, the CatIBs retained their activity after three months at 4 °C. In azo dye decolorization assays with Eriochrome Black T and Congo Red, Lac-40EL CatIBs outperformed Lac-17, particularly in the presence of redox mediators.

**Conclusions:**

This work shows that rational peptide design can improve enzyme immobilization, activity, and stability through CatIBs formation. The resulting CatIBs self-assembled efficiently, were easily recovered, and demonstrated operational stability, supporting their potential for scalable production and novel applications in textile wastewater treatment.

**Supplementary Information:**

The online version contains supplementary material available at 10.1186/s12934-026-02949-4.

## Highlights

- Self-forming catalytic inclusion bodies were obtained, guided by a fusion peptide, which enhances the aggregate’s enzymatic activity.
- Stable laccase catalytic inclusion bodies enabled efficient decolorization of azo dyes, supporting their potential for scalable wastewater treatment.
- A bioprocess integrating static cultivation and fusion-driven assembly enables copper-loaded, catalytically active laccase aggregates.

**Supplementary Information:**

The online version contains supplementary material available at 10.1186/s12934-026-02949-4.

## Introduction

The mining, agriculture, livestock, and industrial sectors contribute to pollution [1, 2], by releasing waste that negatively impacts ecosystems [3, 4]. Pollution causes diseases and premature deaths, including lung cancer, asthma, reproductive and neuronal disorders [5–9]. Over 80% of the world’s wastewater is discharged untreated [10]. For example, textile wastewater accounts for 20% of water pollution, with about 50,000 tons of dyes released into the environment annually [11, 12]. For nearly a century, the use of enzymes for wastewater treatment has been proposed [13]. Enzymes have garnered attention due to their efficiency, specificity, and versatility compared to traditional chemical catalysts [14]. Oxidoreductases and hydrolases represent the main enzymes employed in the transformation of contaminants. Oxidoreductases can degrade complex substrates and catalyze the polymerization of simple compounds into polymeric products, often insoluble and challenging for organisms to assimilate [3, 15]. Among the oxidoreductases used in contaminant degradation, there are laccases, oxygenases, and peroxidases. Laccases (benzenediol: oxygen oxidoreductases, EC 1.10.3.2) represent the largest subgroup of blue multicopper oxidases (MCOs) [16, 17]. Because laccases use molecular oxygen as the final electron acceptor and water is generated as a by-product of the reaction, these enzymes are considered environmentally friendly biocatalysts with great biotechnological interest [18, 19]. These enzymes are found in both eukaryotes and prokaryotes, exhibiting functions such as cross-linking monomers, polymer degradation, and ring cleavage in aromatic compounds, among others [16, 17, 19–21].

Bacterial laccases are versatile enzymes that are typically stable and active across diverse conditions and substrates. This makes them valuable for applications such as bioremediation and biotransformation [17, 21, 22]. One potential use for this type of enzyme is treating effluents and breaking down pollutants such as azo and anthraquinone dyes present in textile wastewater, phenolic compounds like parabens and bisphenol A, and emerging contaminants such as antibiotics, hormones, anti-inflammatories, pesticides, and herbicides [22–25].

Among the advantages of bacterial laccases over fungal laccases is their ability to remain active in alkaline media and high salt concentrations [26]. These characteristics make them suitable for application in bioremediation, since these conditions are common in industrial waste, such as that from the textile industry. However, the limited use of these enzymes in bioremediation is due to the high cost associated with their biotechnological recombinant production, processing, stabilization, and purification [27]. Enzyme immobilization is commonly employed to improve operational stability, reusability, and recovery. Although the immobilization process can be costly, it may ultimately reduce overall expenses compared to using free enzymes, particularly in repeated-use or continuous systems [27].

In particular, different CotA laccases from *Bacillus* species have been used in biofuel cells, dye degradation, and lignocellulose modification, among other applications [26]. However, large-scale production is limited because CotA is spore-bound, resulting in low enzyme expression and challenging its production and purification, requiring harsh extraction and cell-disruption steps that often lead to low recovery rates [26, 28, 29]. Since the CotA expression in *Bacillus* occurs primarily during sporulation, resulting in restricted temporal expression [30], recombinant systems, particularly *E. coli*, have become the hosts of choice for CotA production due to their rapid growth, well-defined genetics, and ease of large-scale fermentation [26, 29–33]. Although other expression systems have also been used [34–36]. Soluble bacterial laccases produced in *E. coli*, such as CotA from *B. subtilis*, exhibit decreased activity due to only 1.3 copper atoms per active site, compared to 4 in the native form [28]. By improving culture strategies, such as microaerobic conditions, recombinant CotA could be fully loaded with copper and become active [37]. Recombinant active laccases, such as CotA laccase from *B. subtilis* WD23, produced in *E. coli* BL21(DE3), yield an active enzyme capable of decolorizing Remazol Brilliant Blue R, and Congo Red without mediators [38]. The recombinant CotA from *Bacillus licheniformis* ANSB821, produced in *E. coli* Rosetta (DE3), can transform aflatoxin B1 [39], degrade polluting dyes or decouple phenolic azo dyes in the decolorization of simulated textile effluent [29], or target tetracycline and ampicillin [40]. The recombinant CotA from *Bacillus* sp. PAMC28748, isolated from Antarctica and produced in *E. coli* C41(DE3), was effective in decolorizing Crystal violet, which requires the presence of ABTS, and Coomassie Brilliant Blue without the need for a redox mediator [41]. Furthermore, the *B. clausii* KSM-K16 laccase, an ortholog of CotA from *B. subtilis*, was expressed in *E. coli* BL21(DE3) in a soluble form [26]. This *B. clausii* KSM-K16 CotA is an interesting alkali-adapted laccase that shows enhanced oxidative activity at high pH, increased salt tolerance, and greater stability under alkaline conditions compared to its *B. subtilis* ortholog, also recombinantly expressed [26, 29]. This robustness probably comes from two substitutions (S427Q and V110E) that may reduce anion–copper interactions, suggesting a trade-off between lower catalytic efficiency and resistance to more extreme conditions [26]. Although CotA laccase from *B. clausii* has a shorter thermal half-life at 80 °C than that of *B. subtilis* CotA, it maintains activity under simultaneous high ionic strength and high pH conditions, in which different alkaline or halotolerant laccases begin to lose functionality. For example, the laccases from *Pseudomonas extremorientalis* or *Streptomyces* species are either strongly halotolerant or highly alkali-resistant, they do not match dual robustness [33–35, 42]. Likewise, thermo-alkaline enzymes such as the SN4 laccase from *Bacillus tequilensis* exhibit exceptional thermal stability but lack salt tolerance at alkaline pH [43]. In all cases, the functional characterization was performed using only the enzyme fraction obtained in soluble form.

Heterologous expression of proteins in *E. coli*, driven by strong promoters and high synthesis rates, can lead to the formation of insoluble inclusion bodies (IBs) [44–63], such as the recombinant production of CotA in *E. coli* often results in the formation of inactive IBs and low productivity [26, 28]. The traditional way of observing IBs often overlooks the fact that some of these aggregates may still be active; they are not merely inactive but somewhat structured, as proteinaceous nanomaterials that retain enzymatic activity. These so-called catalytically active IBs (CatIBs) have been engineered to combine high stability, reusability, and functional performance [48–57]. Some examples of enzymes that have been obtained as CatIBs are β-lactamase, endoglucanase D, human dihydrofolate reductase (hDHFR), β-galactosidase, and asparaginase II [47, 53–63]. Moreover, some studies have focused on promoting protein aggregation by fusing them to different aggregation domains [49, 50, 63]. An example is the β-galactosidase fused to the viral capsid protein VP1 of the foot-and-mouth disease virus, which yields CatIBs that show at least 66% greater activity than their soluble counterparts [47, 64, 65]. Furthermore, some peptides of varying lengths can aid in the self-assembly of the proteins of interest [63, 66–70]. The peptides may have amphipathic properties, enabling them to self-associate and leading to aggregation of the fusion protein [63, 69, 70].

Here, we produced and characterized two laccase enzymes related to those from *Bacillus clausii* KSM-K16, which were heterologously expressed in *E. coli* BL21 (DE3) and obtained as CatIBs. To improve the formation of designed catalytic aggregates, a fusion peptide like one previously reported to promote protein self-assembly was incorporated into one of the laccase variants. Building on this strategy, we aimed to exploit peptide-guided aggregation to produce laccases directly as CatIBs, facilitating their self-assembly into catalytically active structures. We assessed protein yields, enzymatic activities, and several properties of the CatIBs, including their storage stability. Furthermore, their application in decolorizing azo dyes, specifically Congo Red and Eriochrome Black T, was evaluated. To our knowledge, this is the first study to report catalytically active laccase proteins in active nanostructured protein aggregates.

## Materials and methods

### Design, construction of coding sequences, plasmids, and bacterial strains

The coding sequences selected encode a laccase enzyme (CotA) from *Shouchella clausii*, formerly known as *B. clausii* (WP_011247492.1). The gene encoding the laccase, designated as Lac-17, contains an extended open reading frame that encodes two additional amino acids at the C-terminus (Glu and Leu) as a consequence of the gene construction strategy, as a cloning scar. In the fusion construct Lac-40EL, the open reading frame of *cotA* is further modified to encode an extra 35-amino-acid peptide at the C-terminus, designed to promote protein aggregation and assist the formation of IBs. All the synthesized sequences were optimized for *E. coli* preferential codon usage [71]. The resulting nucleotide sequences were chemically synthesized (GenScript Biotech, USA), and each gene construct was cloned into the pET15b plasmid (Novagen^®^, Germany). Each plasmid was amplified in *E. coli* TOP10 cells (Thermo Fisher Scientific, USA) and purified using the QIAprep^®^ Spin Miniprep kit (Qiagen, Germany). Then, *E. coli* BL21(DE3) cells were transformed, and positive colonies were selected based on their resistance to ampicillin (100 µg/mL). Positive transformants were stored in 30% glycerol at -70 °C [45, 72]. The *E. coli* BL21(DE3) strain was used for Isopropyl β-d-1-thiogalactopyranoside (IPTG)-induced expression of Lac-17 and Lac-40EL without a His-tag.

### Shake flask cultures of *E. coli* producing Lac-17 and Lac-40EL

Bacterial cultures were grown in 250 mL Erlenmeyer flasks (Duran Erlenmeyer flask, USA) containing 50 mL of Luria-Bertani culture media (containing ampicillin at 100 µg/mL), inoculated with 500 µL of the working vials of recombinant *E. coli* BL21(DE3), using handmade plugs from gauze and cotton [73]. Recombinant *E. coli* growth was monitored by measuring optical density (OD) at 600 nm, and OD values were converted to dry cell weight using a previously established linear relationship [72, 73].

The cultures, made at least in triplicate, started at 30 °C and 200 rpm in an orbital shaker (New Brunswick Scientific C251, Eppendorf, USA) with a shaking diameter of 25 mm. When cultures reached values near 0.6 absorbance units (A.U.) measured at 600 nm (Spectronic Genesys 20, Thermo, USA), they were induced with 0.1 mM IPTG (Merck-Millipore, Billerica, MA, USA); simultaneously, 0.25 mM CuSO₄ was added. At this point, the temperature and agitation were reduced to 25 °C and 120 rpm, respectively. After four hours of induction, agitation was stopped until the end of 22-hour cultures [26, 29, 37, 38]. During cultivation, 1 mL samples were taken at various time points, and the biomass was recovered by centrifugation at 11,800 × *g* for 10 min at 10 °C. The supernatant was discarded, and the samples were stored at -20 °C [72, 73].

### Protein recovery and quantification

To recover the cytoplasmic proteins, cells were resuspended in 300 µL of lysis buffer (50 mM Tris∙HCl, 100 mM NaCl, and 0.1 mM PMSF, pH = 7) and sonicated in a SoniPrep150 (Sanyo-Gallen-Kamp, UK) with 10 cycles of 30 s pulses at 10% amplitude [45, 72, 73]. To characterize the protein content, the soluble protein extracts were separated from insoluble protein (IP) fractions by centrifugation at 11,800 × *g*. To identify the protein profile by SDS-PAGE, total and IP fractions were dissolved in IEF buffer (7 M Urea, 2 M Thiourea, 40 mM DTT, and 2% w/v CHAPS). All fractions were analyzed by SDS-PAGE (15%) under denaturing conditions and stained with Coomassie Brilliant Blue G-250 [45, 72, 73]. The soluble fraction and IBs were stored at -20 °C [49, 50, 57] and subsequently used to determine enzymatic activity.

Protein concentration was determined using the Bradford method with Bio-Rad reagents and a Stat-Fax 4200 plate reader (Awareness Technology, USA), measuring absorbance at 600 nm. The calibration curve was generated using bovine serum albumin (BSA) at 0 to 0.75 mg/mL concentrations. To quantify total protein and IBs were resuspended in IEF buffer for four hours at room temperature. Densitometry of SDS-PAGE gels of the different protein fractions was performed using ImageLab v6.2 software (BioRad, USA).

### Attenuated total Reflection-Fourier transform infrared (ATR-FTIR) spectroscopy of IBs

ATR-FTIR was employed to analyze the secondary structure of IBs formed by Lac17 and Lac-40EL at 3 and 10 h after induction. IBs were previously dried in a SpeedVac Concentrator Savant ISS110 (Thermo Fisher Scientific, USA). Spectra were acquired using a Shimadzu IRAffinity-1 S FTIR spectrometer (Shimadzu, Japan) equipped with a Specac Quest ATR diamond accessory (Specac Limited, UK). IBs were analyzed over a wavenumber range of 1500–1700 cm⁻¹. For each spectrum, 40 interferograms with 2 cm^− 1^ resolution were collected and averaged to improve the signal-to-noise ratio [45, 72, 73]. Second-derivative spectra of the amide I region were calculated using a 13-point smoothing procedure in LabSolutions IR software (Shimadzu) to identify frequencies corresponding to distinct secondary-structure components [72–76]. Prior to comparative analysis, spectra were normalized using the tyrosine band at 1515 cm⁻¹ as an internal reference [45, 72, 73, 77]. ATR-FTIR measurements were performed in triplicate using samples from three independent cultures for each condition.

### IBs image analysis by transmission electron microscopy (TEM)

Sample solutions with concentrations between 0.3 and 0.5 mg/mL were negatively stained using the methodology described in [77]. Samples were applied to EMS carbon-copper grids. Uranyl acetate (2.5% w/v) (Electron Microscopy Science) was used on the grid for 30 s, and the excess stain was blotted directly. Subsequently, the uranyl acetate application was left to dry ON before being mounted in the sample holder. TEM was performed at 80 kV using a ZEISS LIBRA 120 Transmission Electron Microscope. Images were recorded with a GATAN CCD, and the resulting data were analyzed with Digital Micrograph Software (Gatan, Pleasanton, CA).

### Laccase activity: oxidation of ABTS

Assays were performed with either 200 µg of insoluble protein fractions or 10 µg of soluble protein fractions. In these assays, 0.2 mM ABTS served as the substrate, in 500 µL of 100 mM citrate/200 mM phosphate buffer pH 4, corresponding to the pH value of maximum activity for this substrate, and at 25 °C [26]. The reaction progress was monitored by measuring the change in absorbance at 420 nm [26]. The molar absorption coefficient of the ABTS was set to ε420nm = 36,000 M⁻¹ cm⁻¹ [78–80].

### Dye decoloration qualitative assays

To assess the dye-decolorizing capability of recombinant laccase proteins, 200 µL of reaction medium containing 10 µg of insoluble fraction or 1 µg of soluble fraction was added to each well of 96-well plates. The azo dyes evaluated, at a concentration of 200 mg/L, were Eriochrome Black T and Congo Red. The reaction media evaluated were a synthetic textile mixture (pH 8) and a 100 mM Tris buffer (pH 9). Acetosyringone (0.1 mM) was evaluated as a mediator in both reaction media [81]. The synthetic mixture was prepared to simulate the typical chemical profile of textile effluents (pH 8), which contain a high salt load and essential nutrients [82]. The mixture includes (mg/L): dye 200, (NH₄)₂HPO₄ 24, NH₄Cl 31, CaCl₂ 119.5, MgCl₂ 16.5, FeCl₃ 3.5, NaNO₃ 2.7, Na₂SO₄ 2,070, and NaCl 26,000.

The soluble protein of a non-laccase-producing strain (named HCP) and lysis buffer were used as negative controls (C-). The reactions were carried out at room temperature for 12 h. The effect of the treatments was evaluated by spectral scans between 400 and 700 nm, after the samples were previously centrifuged at 11,800 × *g* for 10 min.

### Molecular modeling

Using the amino acid sequence of *B. clausii* laccase CotA, appended with the peptide (40EL), we generated deep multiple sequence alignments with MMseqs2 [83]. Structural models were then generated using the generative model Boltz-1, which integrates a steering mechanism during inference [84]. This approach guides the generative process toward structurally and thermodynamically plausible conformations by incorporating external information based on Boltzmann-type distributions, thereby favoring structures consistent with biophysical principles. Twenty independent models were generated using default parameters without additional restraints. To group the generated models into conformational subfamilies, we applied the NMRCLUST protocol, which is based on an average-linkage clustering algorithm [85].

### Statistical analysis

All experiments were conducted with at least three biological replicates. A two-way ANOVA with Tukey’s test was used for comparing multiple means, except for comparisons of growth rates and histograms of CI diameters, where Student’s t-test and the Kolmogorov-Smirnov test were used, respectively.

## Results

### Expression and growth kinetics of engineered laccase constructs in *E. coli*

Here, we propose a novel strategy to obtain an auto-ensembled and immobilized bacterial laccase in CatIBs, a scalable approach designed to facilitate large-scale water treatment systems. We developed a peptide for fusion with laccase to assist its auto-assembly into nanoparticles. A bacterial laccase protein (CotA) from *B. clausii* was used as a model, which exhibits activity in the presence of up to 1 M NaCl over a broader pH range, essential properties for its utility in extreme environments [26], such as those found in wastewater. Even though the *cotA* gene from *B. clausii* had not been previously characterized in detail, certain features of this Gram-positive bacterium, such as its optimal growth at pH values near 9, suggested the presence of specialized enzymes. In particular, the *B. clausii cotA* was cloned, heterologously expressed, and biochemically characterized, showing oxidative activity shifted toward higher pH values compared to its *B. subtilis* ortholog, as well as enhanced salt tolerance and stability under alkaline conditions, consistent with the growth characteristics of the wild-type organism [26, 86]. The CotA laccase from *B. clausii* closely resembles its *B. subtilis* counterpart, sharing approximately 71% sequence identity and displaying similar physicochemical characteristics, including comparable molecular weights (58.4 vs. 58.3 kDa) and acidic theoretical pI values (5.56 and 6.13, respectively). Both enzymes feature a secondary-structure framework dominated by β-sheets interspersed with flexible coil regions. This combination of β-sheet–driven rigidity and coil-associated adaptability is thought to underlie the thermal stability of CotA, providing structural robustness while allowing the conformational flexibility required for activity at elevated temperatures [86].

The gene sequences synthesized using the preferred codon usage in *E. coli* [87, 88] encoding the laccases Lac-17 and Lac-40EL contained the gene sequence coding for the *B. clausii* laccase. Lac-17 contains the amino acids Glu and Leu at the C-terminal. In contrast, Lac-40EL contains an additional 35-amino-acid peptide called 40EL added at the C-terminal, inspired by the ELK16 peptide, which forms antiparallel β-sheet structures, confirmed by FTIR [89], and joined to a flexible linker.

**Fig. 1:**
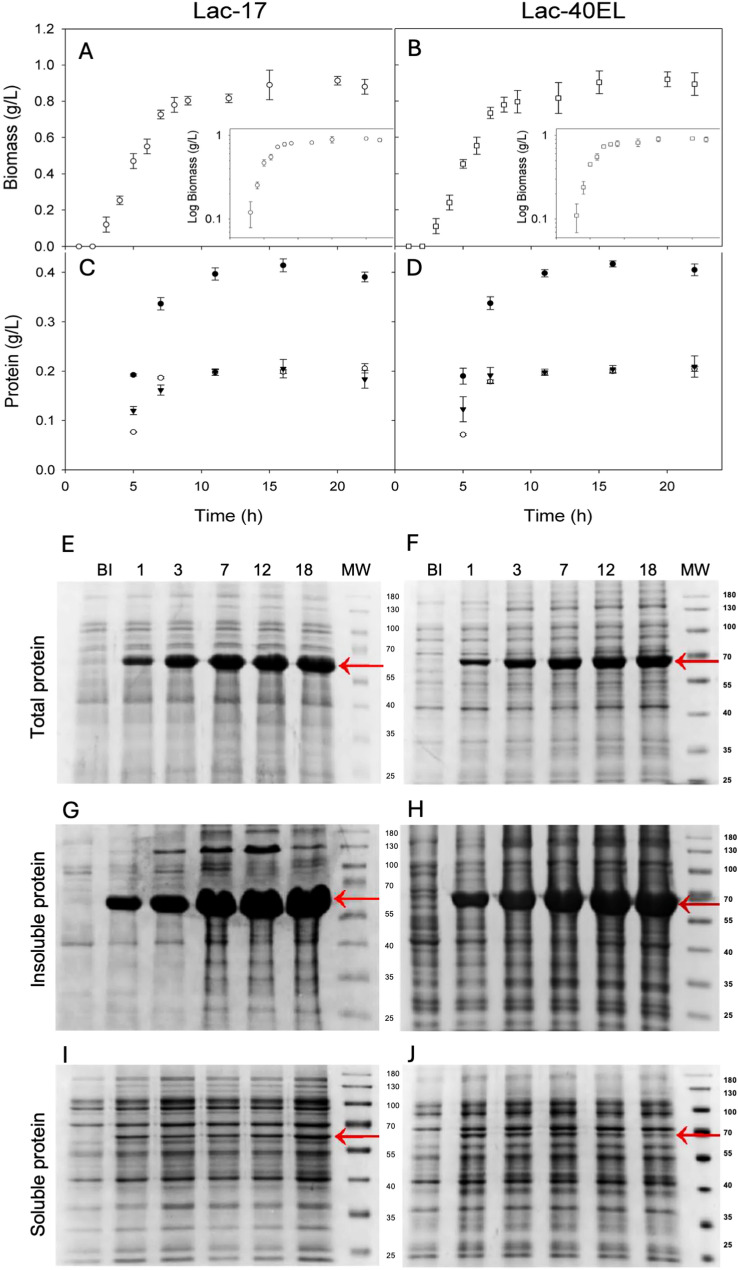
Growth kinetics of *E. coli* Lac-17 (**A**) and *E. coli* Lac-40EL (**B**), on a semilogarithmic scale, are shown in the inset. All cultures were performed in 250 mL shake Erlenmeyer flasks containing 50 mL of LB medium supplemented with ampicillin (100 µg/mL). The culture conditions were modified at specific time points, as indicated by the arrows: (a) induction with 0.1 mM IPTG and addition of 0.25 mM CuSO₄, along with a temperature shift from 30 °C to 25 °C and a reduction in agitation speed from 200 to 120 rpm; and (b) the beginning of a static incubation phase at 25 °C. Kinetic protein accumulation of the total (**closed dots**), insoluble (**open dots**), and soluble protein (**triangles**) in cultures of recombinant strains producing the laccases Lac-17 (**C**) and Lac-40EL (**D**). Protein quantification was performed using the Bradford method, based on measurements from three independent biological replicates. In addition to the determination of total and insoluble protein fractions, proteins were extracted by incubation in 50% IEF buffer for 24 h. Kinetic comparison of protein profiles separated by SDS-PAGE, from the total (**E**,** F**), insoluble (**G**,** H**), and soluble protein (**I**,** J**) accumulated in cultures of recombinant strains producing the laccases Lac-17 (**E**,** G**,**I**) and Lac-40EL (**F**,** H**,**J**). Black arrows indicate the band corresponding to the recombinant laccases. A lane corresponding to the sample collected before induction is presented, followed by lanes representing 1, 3-, 7-, 12-, and 18-hours post-induction. The lane labeled MW corresponds to the molecular weight marker

The recombinant *E. coli* strains producing Lac-17 and Lac-40EL in shake flasks reached similar biomass levels (approximately 0.9 g/L) and showed no significant differences in growth kinetics (Fig. 1A and B). The specific growth rate (µ) of the recombinant strains was determined with values of 0.75 ± 0.04 h^− 1^ before induction. After induction by adding IPTG and CuSO₄, µ was reduced to 0.18 ± 0.01 h^− 1^ for both strains (Table 1). This reduction was likely due to the overexpression of the recombinant protein and the changes from 30 °C to 25 °C and from 200 rpm to 120 rpm, respectively. Following a four-hour induction period, agitation was ceased, thereby reaching the stationary phase. As agitation decreased during the first hours of induction and then ceased, a dissolved oxygen limitation likely occurred, as previously reported under similar culture conditions [73, 90–92]. The oxygen-limited methodology during induction resulted in active laccases, promoting the incorporation of copper into the catalytic site, up to 80 times more at static cultures compared to shaken cultures [31, 37, 93].

**Table 1:** Stoichiometric and kinetic growth parameters for *E. coli* Lac-17 and Lac-40EL strains cultivated at 30 °C and 200 rpm. Once an OD₆₀₀ of ∼0.6 was achieved, expression was induced with 0.1 mM IPTG and 0.25 mM CuSO₄. Subsequently, the temperature and agitation were reduced to 25 °C and 120 rpm, respectively. After a 4-hour induction period, agitation was ceased for the remainder of the cultivation period

Parameter	*E. coli* laccase recombinant protein strain	
	*E. coli* Lac-17	*E. coli* Lac-40EL
^A^µ pre-induction (h^− 1^)	0.75 ± 0.03 ^a^	0.74 ± 0.04 ^a^
^B^µ post-induction (h^− 1^)	0.18 ± 0.01 ^a^	0.18 ± 0.02 ^a^
X_max_ (g/L)	0.91 ± 0.03 ^a^	0.92 ± 0.04 ^a^
^C^Total protein (g/L)	0.41 ± 0.01 ^a^	0.42 ± 0.01 ^a^
^C^Insoluble protein (g/L)	0.21 ± 0.01 ^a^	0.20 ± 0.01 ^a^
^C^Soluble protein (g/L)	0.20 ± 0.02 ^a^	0.20 ± 0.02 ^a^
Y _TP/X_ (g/g)	0.45 ± 0.02 ^a^	0.46 ± 0.01 ^b^
^D^Laccase in insoluble fraction (%)	71 ± 7 ^a^	50 ± 5 ^b^
^D^Laccase in soluble fraction (%)	5 ± 3 ^a^	5 ± 2 ^a^
^E^Laccase in total protein (mg/L)	163 ± 12 ^a^	127 ± 5 ^b^
^E^Laccase in Insoluble fraction (mg/L)	147 ± 4 ^a^	113 ± 5 ^b^
^E^Laccase- in soluble fraction (mg/L)	16 ± 4 ^a^	12 ± 3 ^a^
Y_rLac from total protein /X_	0.19 ± 0.01 ^a^	0.14 ± 0.01 ^b^
Y_rLac from insoluble protein /X_	0.16 ± 0.01 ^a^	0.12 ± 0.01 ^b^

The values presented are the mean and standard deviation derived from three biological replicates per condition. The statistical differences are indicated with different letters (*p* < 0.05)

A: Specific growth rate pre-induction was calculated from the slope of logarithmic growth at 30 °C and 200 rpm in an orbital shaker (New Brunswick Scientific C251, Eppendorf, USA)

B: Specific growth rate post-induction was calculated from the slope of growth after cultures were induced with 0.1 mM Isopropyl β-d-1-thiogalactopyranoside (IPTG), simultaneously with the addition of 0.25 mM CuSO₄ and a reduction to 25 °C and 120 rpm

C: Protein concentration was measured by the Bradford method using Bio-Rad reagent and a Stat-Fax 4200 plate reader at 600 n

E: Recombinant laccase protein in total protein and insoluble fraction (IBs) was calculated by multiplying total protein (g/L) and the percentage of the recombinant band on densitometric analysis identified in SDS-PAGE gels (D) (Fig. 1).

### Total protein and recombinant laccase protein production

During induction, *E. coli* Lac-17 and *E. coli* Lac-40EL accumulated similar volumetric total protein at all times, reaching ∼0.4 g/L after seven hours of induction (Fig. 1C, D; Table 1). From the start of the induction onward, the distribution between soluble and insoluble protein fractions remained relatively constant, with the insoluble fraction representing approximately 50–52% of the total protein (Fig. 1C, D). No significant differences were observed between the *E. coli* Lac-17 and *E. coli* Lac-40EL cultures (Fig. 1C, D). The profiles of total protein solubilized with IEF buffer, along with the soluble and insoluble fractions, were analyzed by SDS-PAGE. They exhibited similar band patterns, with differences in these corresponding to Lac-17 and Lac-40EL (Fig. 1E, F). Lac-17 and Lac-40EL accumulated within the first few hours after culture induction (Fig. 1E, F), predominantly in the insoluble fraction, accounting for ~ 85% at the 22-hour cultivation time point (Fig. 1G, H). In both cases, the amount of soluble recombinant protein was very low, with only faint bands visible on the gel (Fig. 1I, J), indicating limited solubility under the tested expression conditions. These results suggest that both proteins were mainly produced as inclusion bodies (IBs).

**Fig. 2:**
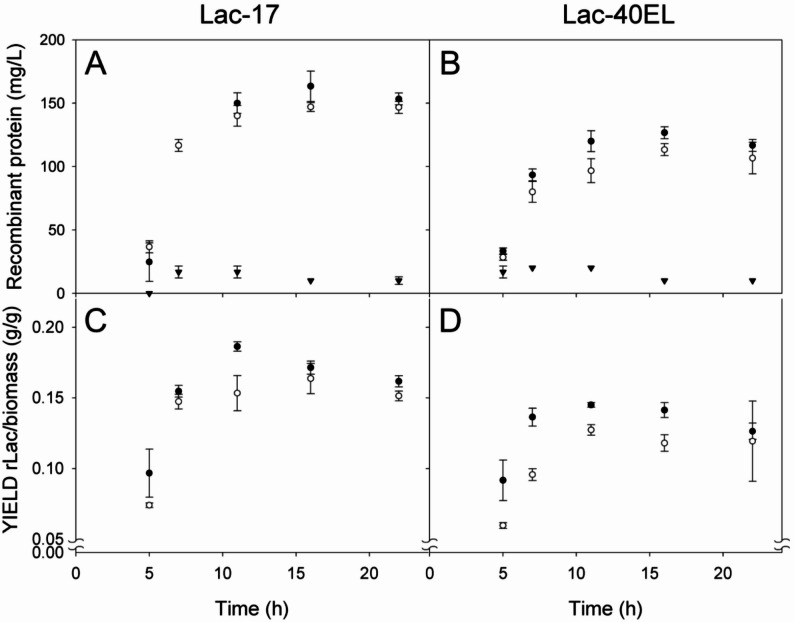
Kinetic protein accumulation of the recombinant laccase proteins Lac-17 (**A**) and Lac-40EL (**B**) on the total (**closed dots**), insoluble (**open dots**), and soluble protein (**triangles**) in cultures. Kinetic yield of the recombinant laccase proteins Lac-17 (**C**) and Lac-40EL (**D**) over biomass on the total (**closed dots**) and insoluble (**open dots**) in cultures. The quantification of the recombinant laccase proteins Lac-17 and Lac-40EL was performed by combining total protein concentration data with densitometric analysis of the SDS-PAGE bands corresponding to each protein

The recombinant laccase proteins Lac-17 and Lac-40EL were quantified by combining total protein concentration data with densitometry of SDS-PAGE bands corresponding to each protein (Fig. 2; Table 1). The IBs containing Lac-17 were almost 20% more enriched in recombinant protein than those composed of Lac-40EL (Table 1). It was observed that *E. coli* Lac-17 accumulated the highest amount of protein, reaching 163 ± 12 mg/L after 7 h of induction (Fig. 2A; Table 1). Meanwhile, *E. coli* Lac-40EL reached 127 ± 5 mg/L after the same induction time (Fig. 2B; Table 1). From 7 h after induction until the end of cultivation, a substantial amount of aggregated protein was detected in the insoluble fraction, with maximal concentrations of 147 ± 4 mg/L for Lac-17 and 113 ± 5 mg/L for Lac-40EL (Fig. 2A and B; Table 1). The recombinant protein concentration in the soluble fraction did not exceed 20 mg/L in either clone (Fig. 2A, B).

In general, the total protein/biomass yield (g/g) (Y_TP/X_) and insoluble protein/biomass yield (g/g) (Y_IP/X_) did not show significant differences (Table 1). Through induction, Lac-17 consistently exhibited a significantly higher total rLac/biomass (g/g) (Y_rLacT/X_ /X) yield than Lac-40EL, averaging 26% more (*p* < 0.05) (Fig. 2C and D). The insoluble rLac/biomass (Y_rLacI/X_) yield of Lac-17 was, on average, 25% higher than that of Lac-40EL after 3 h of induction (Fig. 2C and D). These results indicate differences in recombinant protein accumulation between the two constructs, despite the only variation being the fusion peptide. We suggest that the length and structural characteristics of 40EL peptide extension affect the overall productivity of the recombinant laccase protein in *E. coli*.

### Inclusion bodies characterization

**Fig. 3:**
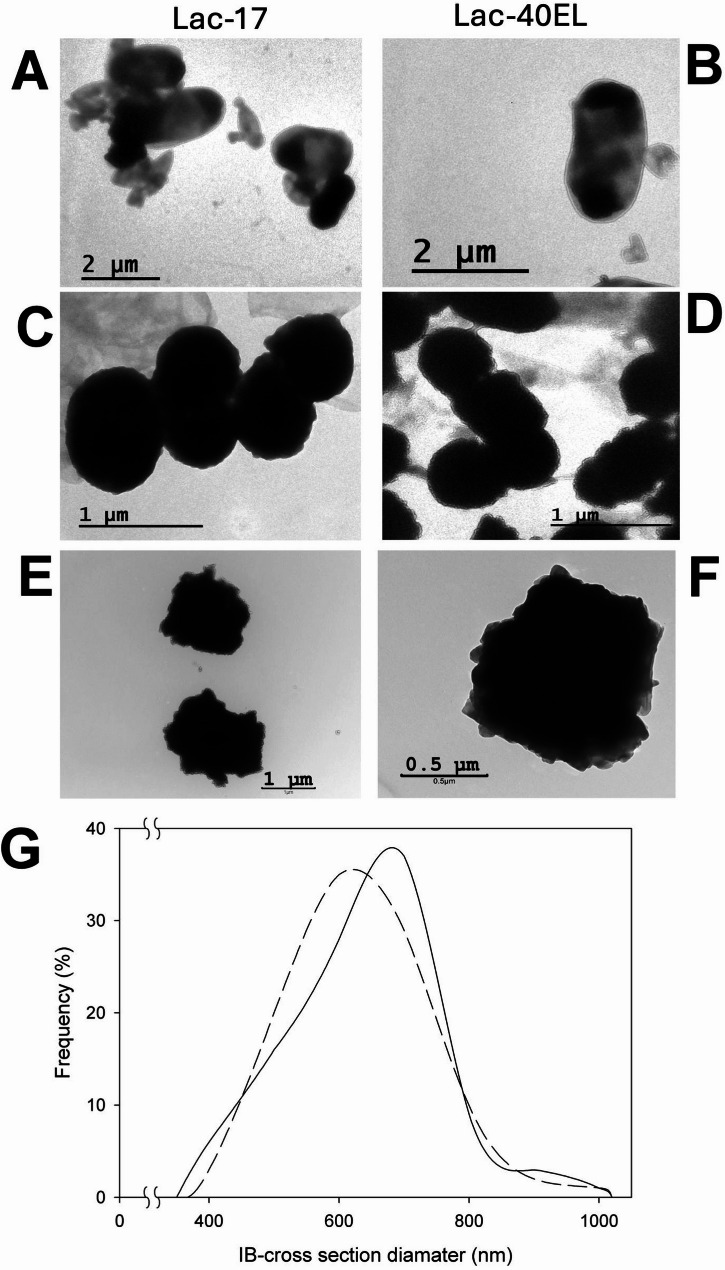
Transmission electron micrographs depict cells containing inclusion bodies (IBs) of laccase proteins and inclusion bodies Lac-17 (**A**) and Lac-40EL (**B**). Isolated IBs of laccase proteins Lac-17 (**C**,** E**) and Lac-40EL (**D**,** F**) are also shown. The bars represent the scale. Histograms (**G**) illustrate the distribution of IBs sizes for laccase proteins Lac-17 (**dashed**) and Lac-40EL (**solid**). The distribution of the mean diameters did not show significant differences in a Kolmogorov–Smirnov test (*p* < 0.05)

The size and structural compactness of catalytic inclusion bodies (CatIBs) can significantly influence their enzymatic activity. Smaller and less densely packed IBs may exhibit a higher surface-to-volume ratio, which can enhance substrate accessibility to catalytically active sites, resulting in increased apparent enzymatic activity [57, 60, 94, 95]. The observed size of Lac-17 and Lac-40EL CatIBs, measured from transmission electron microscopy images taken at the end of the culture, ranged from 400 nm to 900 nm, with most displaying a pseudospherical shape (Fig. 3C, D, E and F). Notably, two IBs per cell were frequently observed (Fig. 3A, B). The histograms show small but no significant differences in sizes observed and the distribution of their average diameters (Fig. 3G). Usually, CatIBs presented a semispherical appearance and seemed to be porous, exhibiting a high degree of hydration [96–98]. In Fig. 3, the dense appearance of the IBs may be caused by the high copper content in the recombinant laccase proteins.

### Laccase activity in soluble and insoluble protein fractions

**Fig. 4:**
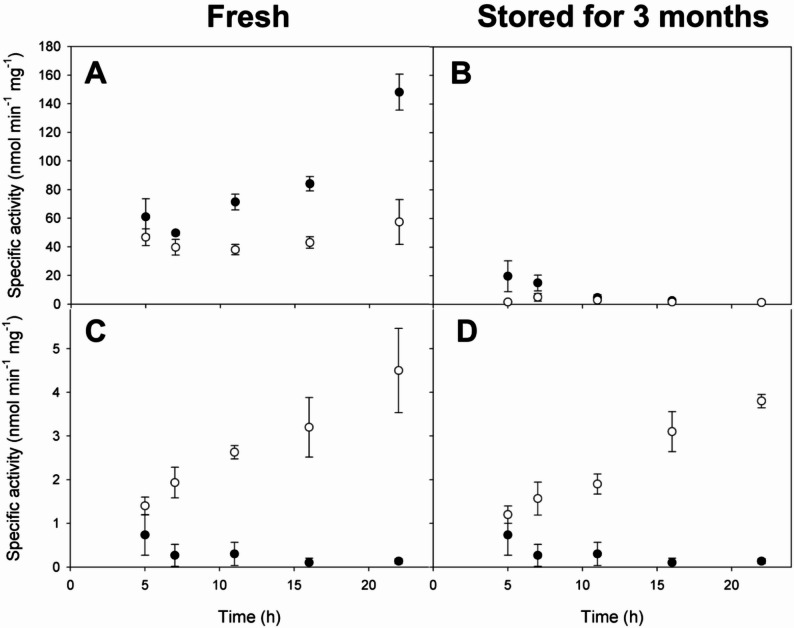
Comparison of the specific activity of Lac-17 (**closed dots**) and Lac-40EL (**open dots**) in soluble (**A**,** B**) and insoluble (inclusion bodies) fractions (**C**,** D**) from freshly prepared samples (**A**,** C**) and samples stored for 3 months (**B**,** D**). The assays were performed using 200 µg of insoluble protein or 10 µg of soluble protein, extracted from cultures at different time points following induction with 0.1 mM IPTG and the addition of 0.25 mM CuSO_4_. Reactions were performed in triplicate with stirring at 25 °C, using a reaction volume of 1 mL with 0.2 mM ABTS in citrate/phosphate buffer (pH 4), and quantified at 420 nm

To assess the impact of the fusion peptide on the enzymatic activity of the recombinant Lac-17 and Lac-40EL, in both their soluble and insoluble forms, we measured the specific activity of each using ABTS as a substrate. This approach also provides indirect insight into the influence of fusion tags on the overall protein conformation. We measured the specific activity on freshly extracted samples and after three months of storage (4 °C). The soluble protein fraction from the *E. coli* Lac-17 strain exhibited the highest activity at the end of the culture (22 h of total culture), three times greater than that of Lac-40EL (Fig. 4A), indicating the distinct effects of the fusion tags on the soluble laccase’s activity. Interestingly, the specific activity of Lac-17 on the soluble fraction increased over time (Fig. 4A), even though there was no similar rise in Lac-17 production (Fig. 2A). This rise can indicate an increase in copper loading, essential for structuring the trinuclear site [ [99], . However, the activity of the laccase proteins in the soluble fraction dropped to near-zero after storage for three months at 4 °C (Fig. 4B).

We added a peptide fusion to Lac-40EL to improve ordered protein aggregation, enabling the formation of self-assembled CatIBs that avoid immobilization and purification while preserving catalytic activity. The specific activity of Lac-40EL CatIBs was 16 to 22 times higher than that of Lac-17 CatIBs (Fig. 4C). Despite this, the amount of recombinant Lac-40EL within the CatIBs was approximately 20% lower than Lac-17 (Fig. 2A, B), suggesting that the enhanced activity was not due to increased recombinant protein content but rather to improved catalytic accessibility or folding within the catIB matrix. In contrast to the laccase activity drop in the soluble fraction during shelf life, the enzymatic activity of the laccase protein Lac-40EL within the CatIBs was maintained throughout the three-month storage period at 4 °C (Fig. 4D).

Increased activity in Lac-40EL CatIBs could be associated with the enrichment of native conformers and active structures in the aggregates, possibly related to the designed aggregation peptide 40EL. This peptide could facilitate the macro-structuring of active catalytic sites [47], even in aggregated proteins within the CatIBs. Until now, no CatIBs with laccase activity have been described, nor has a protocol been established for incorporating copper into CatIBs or fusion proteins.

Although the recovered laccase activity from CatIBs constitutes between 3% and 6% of the activity of soluble Lac-17 (Fig. 4A and C), this discrepancy can be primarily attributed to factors such as diffusional limitations of an immobilized enzyme, as well as other structural and conformational heterogeneities of the CatIBs that impact catalytic efficiency [93]. Since CatIBs are porous materials, they impose limitations on substrate diffusion from the reaction to their surfaces and into their interiors, resulting in a slower substrate conversion rate [97, 100, 101].

### Dye transformation

We assessed the decolorization ability of Lac-17 and Lac-40EL in both soluble and insoluble forms by testing their activity against two representative azo dyes: Eriochrome Black T and Congo Red. These compounds were chosen because of their structural complexity and widespread use, making them suitable models for evaluating the enzyme’s potential for dye-degradation applications.

**Fig. 5:**
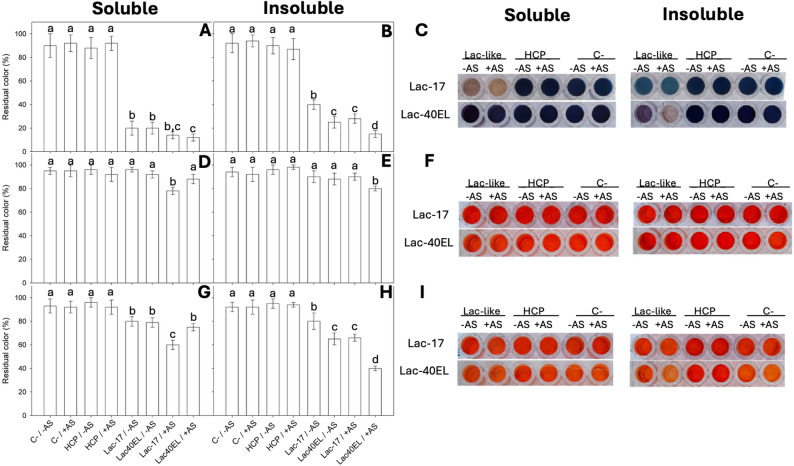
Comparison of Eriochrome Black T degradation in 100 mM Tris pH 9 with Lac-17 and Lac-40EL in soluble (**A**,** C**) and inclusion bodies fractions (**B**,** C**). Comparison of Congo Red degradation in 100 mM Tris pH 9 with Lac-17 and Lac-40EL in soluble (**D**,** F**) and inclusion bodies fractions (**E**,** F**). Comparison of Congo Red degradation in a synthetic mixture pH 8 with Lac-17 and Lac-40EL in soluble (**G, I**) and inclusion bodies fractions (**H, I)**. Reactions were carried out in duplicate in microplates, using a reaction volume of 200 µL with either 1 µg of soluble fraction or 10 µg of IBs. The dye was used at 200 mg/L, and acetosyringone at 0.1 mM. −AS= without addition of acetosyringone, +AS= with addition of acetosyringone. Samples were centrifuged at 11,800 × *g* for 10 min before visual analysis. Quantitative comparisons were made with the absorbance values at 626 nm. C- (untreated samples) are reactions without protein. HCP reactions with equivalent amounts of protein from non-laccase-producing *E. coli*. Significant differences between treatments (*p* < 0.05) are indicated by different lowercase letters above the bars

Lac-40EL displayed an approximately tenfold difference in specific activity between CatIBs and the soluble form in ABTS assays (Fig. 4A and C) at the end of cultures (22 h). To account for this disparity, dye decolorization assays were performed with a tenfold difference in soluble and CatIBs protein amounts. Treatments with the soluble fraction of recombinant laccase proteins in 100 mM Tris at pH 9 showed changes in decolorization for both azo dyes, with and without acetosyringone (AS) (Fig. 5A and F). For Eriochrome Black T, treatment using Lac-17 and Lac-40EL in soluble fractions resulted in decolorization of approximately 84% and 89%, respectively, without and with the addition of AS (Fig. 5A and C). The transformations caused a reduction in the dark blue of the dye or a shift to a light, translucent brown. Interestingly, when using CatIBs, Lac-40EL was more active on Eriochrome Black T, with decolorizations of around 78% without AS and increasing to 88% with AS (Fig. 5B, C). While the Lac-17 CatIBs were able to decolorize Eriochrome Black T, they achieved around 56% and 71% of the effect, respectively, without and with AS (Fig. 5A and C). Although a color change was detected in the controls containing non-associated proteins (HCP) and in the negative control (C-), it was significantly lower than that observed with the evaluated enzymes (Fig. 5). These results indicate that the fusion with the 40EL peptide had a greater impact on the conformation of the soluble protein, mainly in CatIBs, influencing its enzymatic activity. Upon testing Eriochrome Black T in a synthetic textile mixture at pH 8, precipitation was observed in all samples, including those without enzymatic treatment. Consequently, no comparisons were conducted under these conditions.

Regarding Congo Red in 100 mM Tris pH 9, Lac-17 in soluble fraction showed a significant decolorization of nearly 18% in the presence of AS (Fig. 5D). In contrast, Lac-40EL, with or without AS, exhibited no differences compared to the controls (Fig. 5D and F). Lac-40EL CatIBs with AS showed 15% higher conversions than Lac-17 CatIBs and controls (Fig. 5E and F). Using a synthetic textile mixture at pH 8 and Congo Red as a dye, the highest decolorations (44%) were observed with Lac-17 in soluble fractions with AS (Fig. 5G). Meanwhile, Lac-40EL in the insoluble fraction, with and without AS, decolored Congo Red more efficiently than controls (30% and 61%, respectively) (Fig. 5H and I).

### Lac-40EL conformational context

**Fig. 6:**
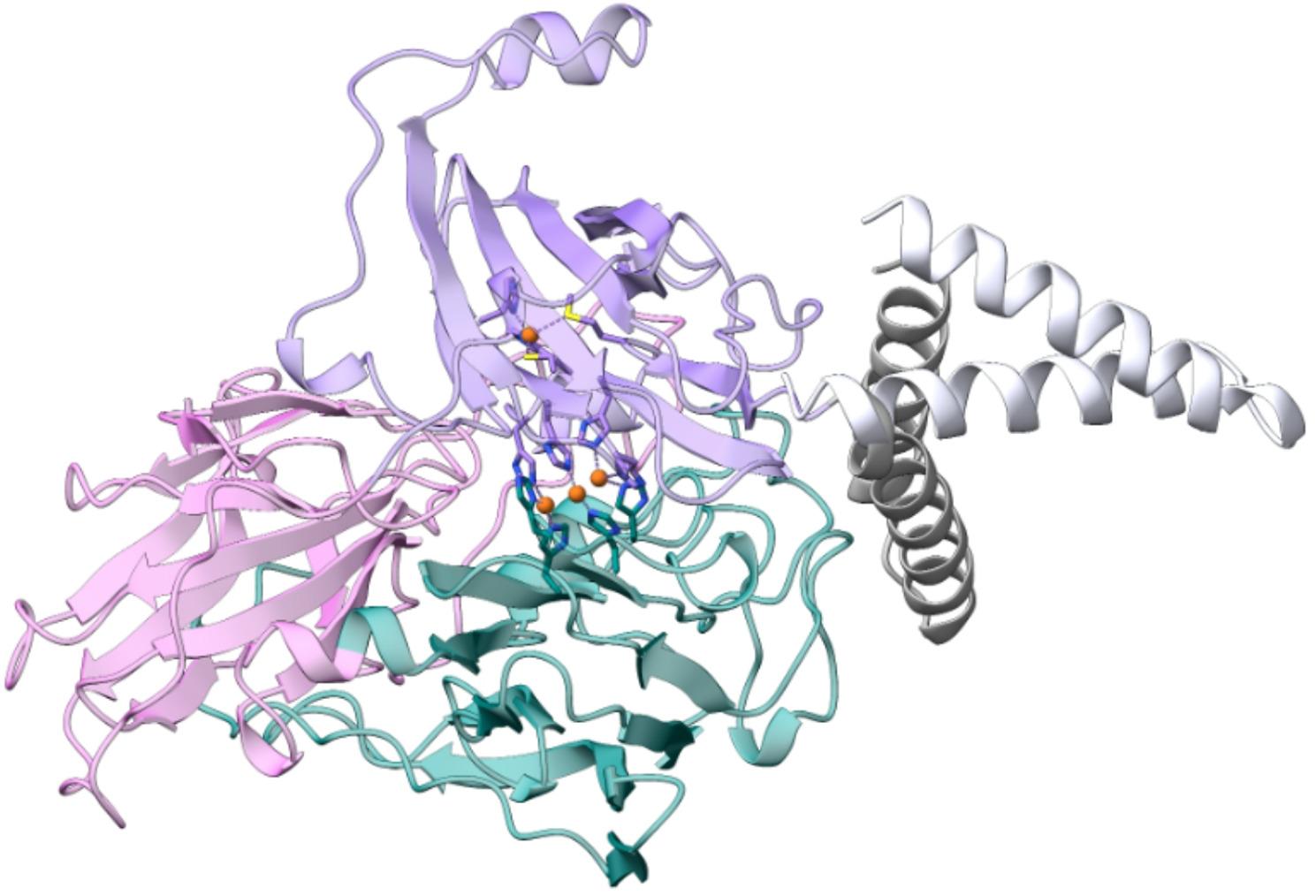
Three-dimensional model of *B. clausii* laccase protein (CotA) fused to the 40-amino-acid peptide at the C-terminus (40EL). The predicted structure of the Lac-40EL includes four copper ion coordination sites, a feature consistent with that experimentally determined structure of *B. subtilis* laccase (PDB ID 1GSK [30]), which shares the highest sequence identity (59.3%) with *B. clausii* laccase among all structurally characterized laccases. The representative centroids of the two clusters identified among the top 20 ranked conformers of the aggregation peptide generated by Boltz-1 are shown

To explore the conformational context of the Lac-40EL chimeric construct of *B. clausii* laccase CotA, which includes a synthetic peptide designed to promote oligomerization and the formation of CatIBs, a structural model was generated using Boltz-1 (Fig. 6), a recently developed AI-based method [84]. The model predicted a typical bacterial laccase architecture, comprising three cupredoxin-like domains, each exhibiting a Greek-key β-barrel motif [22]. These domains were arranged compactly and host the highly conserved type T1 and T2/T3 copper-binding sites. The active site, situated in the central domain, exhibited the expected topology for electron transfer, featuring histidine and cysteine residues that participate in metal coordination and redox catalysis. The predicted fold of *B. clausii* laccase CotA was very similar to the crystallographic structure of its *B. subtilis* ortholog, with a global RMSD of 1.27 Å between the two enzymes. Moreover, the two copper-coordination sites exhibited a nearly perfect overlap, with 100% conservation of the coordinating residues (Fig. S1).

**Fig. 7:**
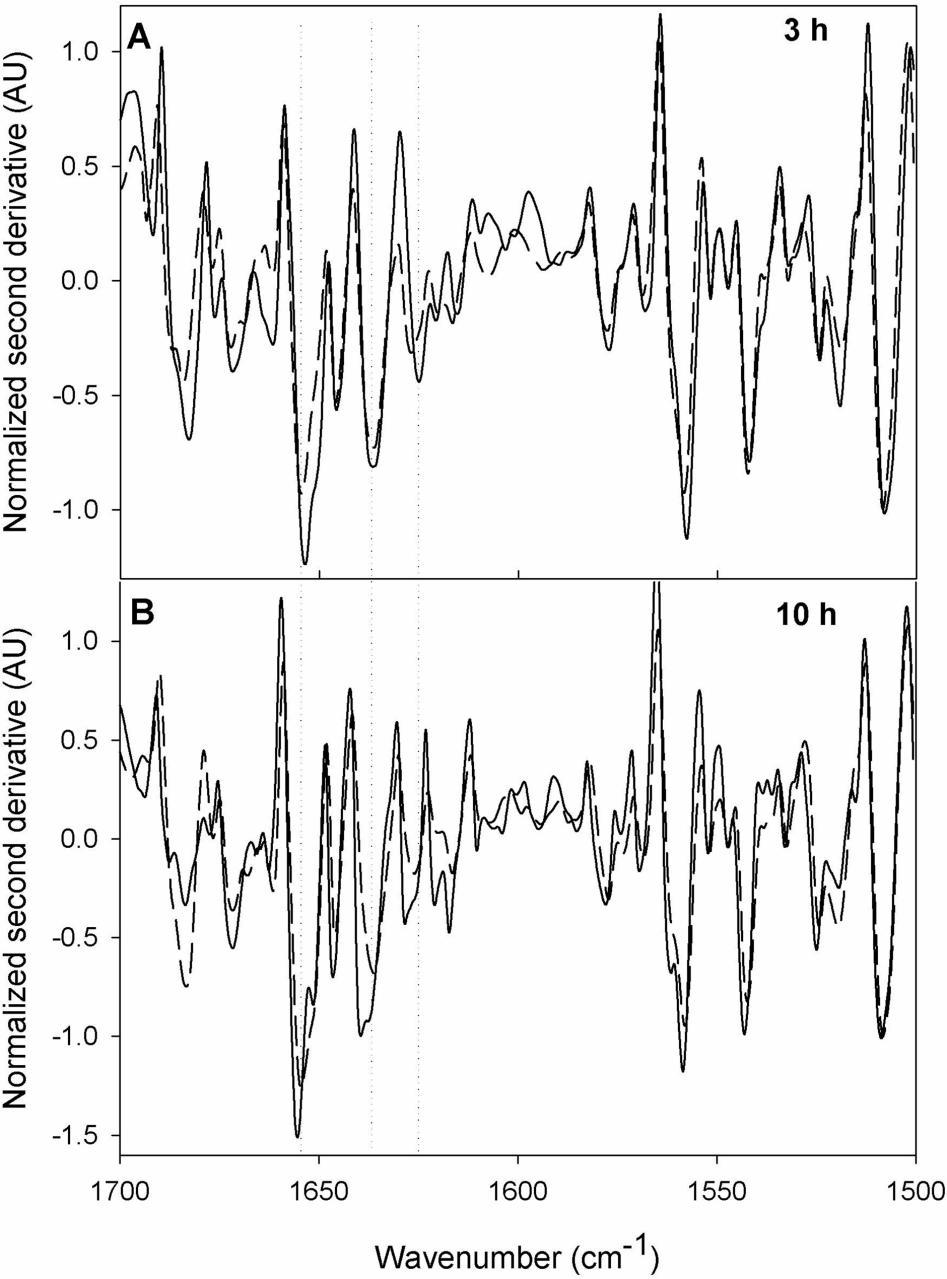
Comparison of FTIR spectra of Lac17 (dashed) and Lac-40EL samples (continuous). FTIR spectra of Lac17 and Lac-40EL were recorded from samples collected 3 h (**A**) and 10 h (**B**) post-induction. Spectra shown correspond to the average of three independent biological replicates. The comparison focuses on differences in the amide I region (1700–1500 cm⁻¹), highlighting variations in secondary-structure features

The 40EL peptide was predicted to adopt an antiparallel α-hairpin structure at its carboxy terminus. A clustering analysis of the 20 highest-scoring models generated by Boltz-1 revealed two main orientations for the fusion peptide. The majority cluster (cluster 1), comprising 13 models, placed the α-helical pair in nonspecific contact with the protein surface. In contrast, the minority cluster (cluster 2) showed the peptide fully exposed to solvent (Fig. 6). Consistent with the Boltz-1 prediction, other algorithms such as AlphaFold [102], RobettaFold [103], and I-TASSER [104] also predict a high helical content in the aggregator peptide fused to the laccase (data not shown). Nevertheless, it is important to note that experimentally, ELK16 alone and joined with amadoriase II or β-xylosidase has been shown to adopt an antiparallel β-strand conformation within IBs [69, 89]. This highlights that the structural conformation that these repetitive peptides adopt within engineered IBs may differ from the in silico prediction, and that current state-of-the-art structure predictors still require refinement to model repetitive aggregation-prone peptides. In any case, these models suggest that the peptide adopts an external conformation projecting from the globular core of the enzyme, without structurally interfering with the active site. This rational design and placement of the fusion peptide at the carboxy terminus of the laccase do not disrupt the catalytic function while promoting the ordered aggregation of the enzyme, consistent with the enzymatic activity retained within the CatIBs observed experimentally. Furthermore, FTIR analysis showed differences in the secondary-structure composition of IBs formed by Lac-17 and Lac-40EL (Fig. 7). After 3 h post-induction, Lac-17 IBs displayed pronounced bands in the amide I region corresponding to β-sheet structures (~ 1636 cm⁻¹) and amyloid-like assemblies (~ 1626 cm⁻¹), together with a detectable contribution from α-helical conformations centered at ~ 1655 cm⁻¹. In contrast, IBs formed during Lac-40EL production exhibited a more prominent α-helical band at ~ 1655 cm⁻¹, accompanied by an increase in the 1626 cm⁻¹ band. A similar trend was maintained at 10 h post-induction, where the second-derivative spectra continued to show a higher relative intensity of the α-helical band, β-sheet, and amyloid-like assemblies in Lac-40EL CatIBs, compared with those formed containing around 70% of Lac-17. These results indicate that, while IBs change over time toward increased β-sheet–rich organization, Lac-40EL CatIBs retain a higher proportion of α-helical structure relative to Lac17.

## Discussion

Biological wastewater treatment methods are sustainable, yet their performance declines under the extreme physicochemical conditions typical of industrial effluents [105]. For example, immobilized whole-cell systems, often stabilized in natural or artificial biofilms, can enhance degradation capacity but face drawbacks such as uncontrollable biofilm growth, mass-transfer limitations, loss of metabolic activity or viability, and high operational costs [105, 106]. Enzymatic strategies offer a more selective and environmentally friendly alternative [27]. However, free enzymes lack the stability required for industrial applications and thus require immobilization. Laccases, as multi-copper oxidases, are particularly attractive because they can oxidize a wide variety of pollutants, including endocrine disruptors, microplastics, and pharmaceuticals, due to their ability to perform single-electron oxidation of substrates [17, 22, 107]. Different laccases have been used to treat hazardous pollutants, such as synthetic dyes [22, 29, 34, 39, 78, 108], remarking their recyclability and increased operational stability by entrapping or immobilizing the enzymes, which allows improving decolorization efficiency [109, 110]. Enzyme immobilization is commonly employed in industrial processes to maintain enzymatic activity and stability over time, though it often requires expensive purification procedures [29, 111]. Soluble enzymes can be immobilized on solid supports for easy recovery with minimal loss of activity. Previous studies have led to various immobilization strategies, including cross-linked enzyme aggregates (CLEAs), adsorption onto carriers, or encapsulation in gels, for laccases, thereby immobilizing these biocatalysts under specific conditions [112–114]. Our approach, based on CatIBs, offers distinct advantages in terms of simplicity, reproducibility, and cost-effectiveness. Unlike conventional immobilization methods, CatIBs are generated directly during recombinant protein expression in *E. coli*, eliminating the need for additional immobilization steps, reagents, or carrier materials [115]– [116]. This self-assembly process reduces downstream processing costs and avoids enzyme inactivation associated with harsh cross-linking or binding conditions. Although bacterial IBs have been viewed as inactive and unfolded proteins produced during overexpression of recombinant genes in bioprocesses and in particular during CotA production [27, 28], they serve as the raw material for extracting soluble proteins since they could trap up to 50% of total protein [49, 50, 60, 117]. Furthermore, IBs can be designed to contain correctly folded proteins. Notably, their self-assembly may mimic the natural immobilization of enzymes, enabling them to retain catalytic activity, thus forming CatIBs [49, 50].

The present study builds upon previous biochemical characterization by engineering and producing a self-assembling variant of the enzyme. We focused on the *B. clausii* CotA, a halotolerant and pH-stable laccase, and we explored its rational modification to promote controlled aggregation into CatIBs, aiming to enhance the activity and stability of recombinant bacterial laccases under alkaline conditions [26, 78, 107]. The interesting CotA laccase from *B. clausii* exhibits better stability at high salt and alkaline pH than CotA from *B. subtilis* [26]. These characteristics could be considered for treating textile effluent wastewater. In this study, the Lac-17 and Lac-40EL variants share 99% and 76% sequence identity, respectively, with CotA from *B. clausii* [26]. In contrast to earlier studies, we omitted a His-tag in our constructs to avoid alterations near the active site that could obscure the effects of our designed mutations. The CotA laccases from *B. clausii* and *B. subtilis* had been previously expressed in *E. coli* BL21(DE3) and characterized in soluble forms [26], without kinetic monitoring of cell growth, nor did it assess the potential effects of sequence differences on host physiology or interest in the laccase aggregate form.

In this work, we propose the engineering of a bacterial laccase protein capable of self-assembling within the host cell, utilizing a novel enzyme immobilization strategy that bypasses the need for purification or chemical modifications. The laccase-producing strains *E. coli* Lac-17 and *E. coli* Lac-40EL showed similar growth patterns and produced comparable amounts of total, soluble, and insoluble proteins (Fig. 1). The Lac-17 and Lac-40EL proteins were mainly expressed as insoluble IBs (Figs. 2 and 3). Furthermore, protein engineering enables the creation of CatIB by fusing aggregation-inducing peptides or protein domains [49, 50, 63]. In this study, it appears that the design of the peptide 40EL resulted in the enzymatic activity of Lac-40EL CatIBs significantly higher than that of Lac-17 CatIBs (Figs. 4 and 5).

Few studies have systematically evaluated the long-term stability of laccases in immobilized systems. Available reports suggest that between 20% and 90% of enzymatic activity can be retained over periods ranging from one to three months, depending on the type of support, immobilization method, and storage conditions [118, 119]. In our case, Lac-40EL CatIBs retained their activity for at least three months, with no statistically significant differences compared to the initial activity when they were produced (Fig. 4). Although soluble Lac-17 showed higher initial enzymatic activity than soluble Lac-40EL, both enzymes lost all activity after three months of storage at 4 °C, highlighting the protective role of CatIBs in maintaining enzymatic function over time (Fig. 4).

The presence of electron-dense aggregates was identified through transmission electron microscopy, suggesting the accumulation of compacted particles with high electron density (Fig. 3) that may be due to increased copper incorporation into the recombinant laccase structure. This effect probably results from the production conditions used, which cause microaerobic environments and enhance copper accumulation in recombinant laccase proteins produced in *E. coli*, as has been suggested [37]. Interestingly, static conditions in submerged cultures of recombinant *E. coli* not only enhance copper incorporation but also appear to facilitate the correct folding and structural maturation of soluble laccase proteins. This is evidenced by the production of catalytically active enzymes such as those from *B. subtilis* 168 and the chimeric laccases derived from *B. amyloliquefaciens* LC02 and *B. subtilis* LS02 [29, 37, 99]. However, these conditions also lead to the aggregation of a substantial amount of protein, largely consisting of disordered recombinant laccases, and are correlated with the low enzymatic activity of Lac-17-derived CatIBs. In contrast, when fused to the 40EL peptide, aggregation appeared to be more directed, promoting the formation of properly folded molecules inside CatIBs. Therefore, this guided aggregation enhanced the recovery of catalytically active and self-assembled IBs. The designed 40EL, inspired by the ELK16 peptide [59, 64, 65, 89], has an amphipathic nature, allowing it self-association [63, 68–70, 89, 120, 121]. It is worth noting that differences in the accumulation of recombinant laccase proteins were observed, with higher levels in Lac-17 cultures (Figs. 2 and 3), the sole difference being the presence of the self-aggregation peptide. This is probably associated with a lower metabolic expenditure, as the Lac-17 protein is smaller, approximately 6 kDa.

Some bacterial laccases have demonstrated potential in decolorizing textile azo dyes [22, 122, 123]. In this study, we observed that CatIBs of Lac-40EL can comprise correctly folded, functional recombinant proteins, as demonstrated by detecting laccase activity, primarily in Lac-40EL in ABTS (Figs. 4 and 5). Nevertheless, the enzymatic activity of soluble Lac-17 was higher than that of soluble Lac-40EL. On the other hand, both laccases (Lac-17 and Lac-40EL), in both crude soluble and insoluble protein fractions, were able to decolorize azo dyes such as Eriochrome Black T and Congo Red (Fig. 5).

In these decolorization assays, higher protein loads were required for the CatIB fractions, reflecting the intrinsic challenge of achieving a high proportion of properly folded, catalytically active protein within CatIBs [49, 51, 52, 60, 95, 97]. Additionally, the lower activity seen in CatIBs compared to the soluble enzyme results from the combined effects of mass-transfer limitations and the conformational heterogeneity intrinsic to immobilized enzyme systems, which together constrain catalytic efficiency [49, 51, 52, 60, 95, 97].

Experimental evidence shows ELK16 peptides form antiparallel β-strand conformations driven by hydrophobic and electrostatic complementarity, particularly within IBs [89]. However, structural modeling predicts the 40EL peptide adopts an antiparallel α-hairpin at the carboxy terminus of CotA (Fig. 6, Fig. S1). Furthermore, FTIR analysis reveals differences in the secondary-structure organization of Lac-17 and Lac-40EL IBs that correlate with their catalytic behavior (Fig. 7). Lac-40EL CatIBs consistently maintain a higher relative presence of α-helical structures compared to Lac-17. Although Lac-40EL IBs display an amyloid-rich architecture, native-like conformations are preserved over time. This organization is characteristic of non-classical CatIBs [94]. It provides a plausible molecular basis for the enhanced catalytic activity in Lac-40EL CatIBs, supporting the role of rational fusion-peptide design in modulating IBs structure toward functional assemblies. In this context, the modeling results and FTIR data converge to indicate that the 40EL peptide promotes ordered aggregation while remaining externally positioned, thereby avoiding interference with the catalytic core.

Alongside the analysis presented, we are developing strategies to improve the catalytic performance *B. clausii* CotA CatIBs. We are also characterizing additional self-assembling CotA orthologs to diversify substrate specificity and optimize enzyme function under the extreme physicochemical conditions expected for integrated water-treatment applications.

## Conclusions

This work presents a novel concept for the production of *Bacillus clausii* CotA laccases as catalytically active inclusion bodies (CatIBs) in recombinant *E. coli*. These self-assembled aggregates retain enzymatic activity and exhibit strong potential for biotechnological applications, particularly in the treatment of textile effluents. While both Lac-17 and its fusion variant Lac-40EL were catalytically active in both soluble and aggregated forms, Lac-40EL CatIBs exhibited the highest activity toward ABTS and superior decolorization of the Eriochrome Black T and Congo Red azo dyes. Given the environmental impact of dyes like Congo Red, these results highlight the importance of utilizing designed and auto-immobilized enzymes as robust biocatalysts for degrading toxic and persistent pollutants. This strategy presents a promising, eco-friendly alternative for the bioremediation of dye-laden wastewater, opening new avenues for developing sustainable enzymatic solutions to industrial pollution.

## Supplementary Information

Below is the link to the electronic supplementary material.

Supplementary Material 1.

## Data Availability

No datasets were generated or analysed during the current study.

## References

[1] Ukaogo PO, Ewuzie U, Onwuka CV. Environmental pollution: causes, effects, and the remedies. In Microorganisms for sustainable environment and health. Elsevier. 2020; pp. 419 – 29.

[2] Harrison S, McAree C, Mulville W, Sullivan T. The problem of agricultural ‘diffuse’ pollution: getting to the point. Sci Total Environ. 2019;677:700–17. 31071672 10.1016/j.scitotenv.2019.04.169

[3] Gianfreda L, Rao MA, Scelza R, De La Luz Mora M. Role of enzymes in environment Cleanup/Remediation. Agro-Industrial wastes as feedstock for enzyme production: apply and exploit the emerging and valuable use options of waste biomass. Elsevier Inc.; 2016.

[4] Karigar CS, Rao SS. Role of microbial enzymes in the bioremediation of pollutants: a review. Enzyme Res. 2011;2011:805187. 21912739 10.4061/2011/805187 PMC3168789

[5] Bai L, Shin S, Burnett RT, Kwong JC, Hystad P, van Donkelaar A, Goldberg MS, Lavigne E, Weichenthal S, Martin RV, Copes R, Kopp A, Chen H. Exposure to ambient air pollution and the incidence of lung cancer and breast cancer in the Ontario population health and environment cohort. Int J Cancer. 2020;146(9):2450–9. 31304979 10.1002/ijc.32575

[6] Frye RE, Cakir J, Rose S, Delhey L, Bennuri SC, Tippett M, Melnyk S, James SJ, Palmer RF, Austin C, Curtin P, Arora M. Prenatal air pollution influences neurodevelopment and behavior in autism spectrum disorder by modulating mitochondrial physiology. Mol Psychiatry. 2021;26(5):1561–77. 32963337 10.1038/s41380-020-00885-2 PMC8159748

[7] Holst GJ, Pedersen CB, Thygesen M, Brandt J, Geels C, Bønløkke JH, Sigsgaard T. Air pollution and family related determinants of asthma onset and persistent wheezing in children: nationwide case-control study. BMJ. 2020;370:m2791. 32816747 10.1136/bmj.m2791 PMC7437497

[8] Gray LE Jr, Ostby J, Ferrell J, Sigmon R, Cooper R, Linder R, Rehnberg G, Goldman J, Laskey J. Correlation of sperm and endocrine measures with reproductive success in rodents. Prog Clin Biol Res. 1989;302:193–206. 2666989

[9] Hernández-Zamora M, Martínez-Jerónimo F, Cristiani-Urbina E, Cañizares-Villanueva RO. Congo red dye affects survival and reproduction in the Cladoceran *Ceriodaphnia dubia*. Effects of direct and dietary exposure. Ecotox. 2016;25(10):1832–40. 10.1007/s10646-016-1731-x 27670667

[10] UNESCO The United Nations world water development report 2017: wastewater: the untapped resource; facts and figures. In Paris, France. 2017;p.202.

[11] Dihom HR, Al-Shaibani MM, Mohamed RMSR, Al-Gheethi AA, Sharma A, Khamidun MHB. Photocatalytic degradation of disperse Azo dyes in textile wastewater using green zinc oxide nanoparticles synthesized in plant extract: A critical review. J Water Process Eng. 2022:47102705.

[12] Knežević N, Vuksanović MM, Banjanac K, Pantić K, Veličković Z, Cvijetić I, Marinković A, Milošević M. Cationic waste hemp fibers-based membrane: case study of anionic pollutants removal through environmentally friendly processes. J Environ Manage. 2024;371:123174. 39504666 10.1016/j.jenvman.2024.123174

[13] Aitken MD. Waste treatment applications of enzymes: opportunities and Obstacles. Chem Eng J. 1993;52(2):49–58.

[14] Thakur M, Medintz IL, Walper SA. Enzymatic bioremediation of organophosphate Compounds—Progress and remaining challenges. Front Bioeng Biotechnol. 2019;7:289. 31781549 10.3389/fbioe.2019.00289 PMC6856225

[15] Kumar A, Gudiukaite R, Gricajeva A, Sadauskas M, Malunavicius V, Kamyab H, Sharma S, Sharma T, Pant D. Microbial lipolytic enzymes – promising energy-efficient biocatalysts in bioremediation. Energy. 2020;192:116674.

[16] Giardina P, Faraco V, Pezzella C, Piscitelli A, Vanhulle S, Sannia G. Laccases: a never-ending story. Cell Mol Life Sci. 2010;67(3):369–85. 19844659 10.1007/s00018-009-0169-1 PMC11115910

[17] Rajendran S, Kalairaj A, Senthilvelan T. A comprehensive review on enzymatic decolorization of various Azo dyes using laccase for the abatement of industrial pollution. Biomass Conv Bioref. 2025;15:13079–101.

[18] Mot AC, Silaghi-Dumitrescu R, Laccases. Complex architectures for one-electron oxidations. Biochem (Moscow). 2012;77(12):1395–07. 10.1134/S0006297912120085 23244736

[19] Janusz G, Pawlik A, Świderska-Burek U, Polak J, Sulej J, Jarosz-Wilkołazka A, Paszczyński A. Laccase Properties, physiological Functions, and evolution. Int J Mol Sci. 2020;21(3):966. 32024019 10.3390/ijms21030966 PMC7036934

[20] Dwivedi UN, Singh P, Pandey VP, Kumar A. Structure–function relationship among bacterial, fungal and plant laccases. J Mol Catal B: Enzym. 2011;68(2):117–28.

[21] Mate DM, Alcalde M. Laccase engineering: from rational design to directed evolution. Biotechnol Adv. 2015;33(1):25–40. 25545886 10.1016/j.biotechadv.2014.12.007

[22] Arregui L, Ayala M, Gómez-Gil X, Gutiérrez-Soto G, Hernández-Luna CE, Herrera de Los Santos M, Levin L, Rojo-Domínguez A, Romero-Martínez D, Saparrat MCN, Trujillo-Roldán MA, Valdez-Cruz NA. Laccases: structure, function, and potential application in water bioremediation. Microb Cell Fact. 2019;18(1):200. 31727078 10.1186/s12934-019-1248-0 PMC6854816

[23] Nunes CS, Kunamneni A. Laccases—properties and applications. Enzymes in human and animal nutrition. Elsevier Inc; 2018. pp. 133–61.

[24] Zhang W, Zhang Z, Ji L, Lu Z, Liu R, Nian B, Hu Y. Laccase immobilized on nanocomposites for wastewater pollutants degradation: current status and future prospects. Bioprocess Biosyst Eng. 2023;46:1513–31. 37458833 10.1007/s00449-023-02907-z

[25] Sutaoney P, Pandya S, Gajarlwar D, Joshi V, Ghosh P. Feasibility and potential of laccase-based enzyme in wastewater treatment through sustainable approach: A review. Environ Sci Pollut Res. 2022;29:86499–527. 10.1007/s11356-022-21565-4 35771325

[26] Brander S, Mikkelsen JD, Kepp KP. Characterization of an alkali- and halide-resistant laccase expressed in *E. coli*: CotA from *Bacillus Clausii*. PLoS ONE. 2014;9(6):e99402. 24915287 10.1371/journal.pone.0099402 PMC4051777

[27] Rodríguez-Couto S. Immobilized-laccase bioreactors for wastewater treatment. Biotechnol J. 2024;19(1):e2300354. 37750809 10.1002/biot.202300354

[28] Martins LO, Soares CM, Pereira MM, Teixeira M, Costa T, Jones GH, Henriques AO. Molecular and biochemical characterization of a highly stable bacterial laccase that occurs as a structural component of the *Bacillus subtilis* endospore coat. J Biol Chem. 2002;277(21):18849–59. 11884407 10.1074/jbc.M200827200

[29] Wang TN, Zhao M. A simple strategy for extracellular production of CotA laccase in *Escherichia coli* and decolorization of simulated textile effluent by Recombinant laccase. Appl Microbiol Biotechnol. 2017;101(2):685–96. 27738721 10.1007/s00253-016-7897-6

[30] Enguita FJ, Martins LO, Henriques AO, Carrondo MA. Crystal structure of a bacterial endospore coat component. A laccase with enhanced thermostability properties. J Biol Chem. 2003;278(21):19416–25. 12637519 10.1074/jbc.M301251200

[31] Ihssen J, Reiss R, Luchsinger R, Thöny-Meyer L, Richter M. Biochemical properties and yields of diverse bacterial laccase-like multicopper oxidases expressed in *Escherichia coli*. Sci Rep. 2015;5:10465. 26068013 10.1038/srep10465 PMC4464401

[32] Skálová T, Dohnálek J, Østergaard LH, Østergaard PR, Kolenko P, Dusková J, Stepánková A, Hasek J. The structure of the small laccase from *Streptomyces coelicolor* reveals a link between laccases and nitrite reductases. J Mol Biol. 2009;385(4):1165–78. 19063896 10.1016/j.jmb.2008.11.024

[33] Zovo K, Pupart H, Van Wieren A, Gillilan RE, Huang Q, Majumdar S, Lukk T. Substitution of the methionine axial ligand of the T1 Copper for the Fungal-like phenylalanine ligand (M298F) causes local structural perturbations that lead to thermal instability and reduced catalytic efficiency of the small laccase from *Streptomyces* Co. ACS Omega. 2022;7(7):6184–94. 35224382 10.1021/acsomega.1c06668 PMC8867573

[34] Dubé E, Shareck F, Hurtubise Y, Daneault C, Beauregard M. Homologous cloning, expression, and characterisation of a laccase from *Streptomyces coelicolor* and enzymatic decolourisation of an Indigo dye. Appl Microbiol Biotechnol. 2008;79(4):597–603. 18437373 10.1007/s00253-008-1475-5

[35] Yadav D, Ranjan B, Mchunu N, Roes-Hill ML, Kudanga T. Secretory expression of Recombinant small laccase from *Streptomyces coelicolor* A3(2) in *Pichia pastoris*. Int J Biol Macromol. 2018;108:642–9. 29203348 10.1016/j.ijbiomac.2017.11.169

[36] Yadav D, Ranjan B, Mchunu N, Le Roes-Hill M, Kudanga T. Enhancing the expression of Recombinant small laccase in *Pichia pastoris* by a double promoter system and application in antibiotics degradation. Folia Microbiol. 2021;66(6):917–30. 34216383 10.1007/s12223-021-00894-w

[37] Durão P, Chen Z, Fernandes AT, Hildebrandt P, Murgida DH, Todorovic S, Pereira MM, Melo EP, Martins LO. Copper incorporation into Recombinant CotA laccase from *Bacillus subtilis*: characterization of fully copper loaded enzymes. J Biol Inorg Chem. 2008;13(2):183–93. 17957391 10.1007/s00775-007-0312-0

[38] Wang C, Cui D, Lu L, Zhang N, Yang H, Zhao M, Dai S. Cloning and characterization of CotA laccase from *Bacillus subtilis* WD23 decoloring dyes. Ann Microbiol. 2016;66(1):461–7.

[39] Guo Y, Qin X, Tang Y, Ma Q, Zhang J, Zhao L. CotA laccase, a novel aflatoxin oxidase from *Bacillus licheniformis*, transforms aflatoxin B1 to aflatoxin Q1 and epi-aflatoxin Q1. Food Chem. 2020a;325:126877. 32387986 10.1016/j.foodchem.2020.126877

[40] Zhang C, You S, Zhang J, Qi W, Su R, He Z. An effective in-situ method for laccase immobilization: excellent activity, effective antibiotic removal rate and low potential ecological risk for degradation products. Bioresour Technol. 2020;308:123271. 32247949 10.1016/j.biortech.2020.123271

[41] Karmacharya J, Shrestha P, Han SR, Lee JH, Oh TJ. Exploiting CotA laccase from Antarctic *Bacillus sp.* PAMC28748 for efficient mediator-assisted dye decolorization and ABTS regeneration. Chemosphere. 2025;372:144137. 39848057 10.1016/j.chemosphere.2025.144137

[42] Neifar M, Chouchane H, Mahjoubi M, Jaouani A, Cherif A. *Pseudomonas extremorientalis* BU118: a new salt-tolerant laccase-secreting bacterium with biotechnological potential in textile Azo dye decolourization. 3 Biotech. 2016;6(1):107. 28330177 10.1007/s13205-016-0425-7 PMC4835423

[43] Sondhi S, Sharma P, Saini S, Puri N, Gupta N. Purification and characterization of an extracellular, thermo-alkali-stable, metal tolerant laccase from *Bacillus tequilensis* SN4. PLoS ONE. 2014;9(5):e96951. 24871763 10.1371/journal.pone.0096951 PMC4037180

[44] Sørensen HP, Mortensen KK. Soluble expression of Recombinant proteins in the cytoplasm of *Escherichia coli*. Microb Cell Fact. 2005;4(1):1. 15629064 10.1186/1475-2859-4-1 PMC544838

[45] Calcines-Cruz C, Olvera A, Castro-Acosta RM, Zavala G, Alagón A, Trujillo-Roldán MA, Valdez-Cruz NA. Recombinant-phospholipase A2 production and architecture of inclusion bodies are affected by pH in *Escherichia coli*. Int J Biol Macromol. 2018;108:826–36. 10.1016/j.ijbiomac.2017.10.178. 29101045 10.1016/j.ijbiomac.2017.10.178

[46] Wurm DJ, Quehenberger J, Mildner J, Eggenreich B, Slouka C, Schwaighofer A, Wieland K, Lendl B, Rajamanickam V, Herwig C, Spadiut O. Teaching an old pET new tricks: tuning of inclusion body formation and properties by a mixed feed system in *E. coli*. Appl Microbiol Biotechnol. 2018;102(2):667–76. 10.1007/s00253-017-8641-6. 29159587 10.1007/s00253-017-8641-6 PMC5756567

[47] García-Fruitós E, González-Montalbán N, Morell M, Vera A, Ferraz RM, Arís A, Ventura S, Villaverde A. Aggregation as bacterial inclusion bodies does not imply inactivation of enzymes and fluorescent proteins. Microb Cell Fact. 2005;4:27. 16156893 10.1186/1475-2859-4-27 PMC1224866

[48] Nahalka J, Nidetzky B. Fusion to a pull-down domain: a novel approach of producing *Trigonopsis variabilis* D-amino acid oxidase as insoluble enzyme aggregates. Biotechnol Bioeng. 2007;97(3):454–61. 17089401 10.1002/bit.21244

[49] Jäger VD, Lamm R, Küsters K, Ölçücü G, Oldiges M, Jaeger KE, Büchs J, Krauss U. Catalytically-active inclusion bodies for biotechnology-general concepts, optimization, and application. Appl Microbiol Biotechnol. 2020;104(17):7313–29. 32651598 10.1007/s00253-020-10760-3 PMC7413871

[50] Jäger VD, Lamm R, Kloß R, Kaganovitch E, Grünberger A, Pohl M, Büchs J, Jaeger KE, Krauss U. A synthetic reaction cascade implemented by colocalization of two proteins within catalytically active inclusion bodies. ACS Synth Biol. 2018;7(9):2282–95. 30053372 10.1021/acssynbio.8b00274

[51] Bello MN, Sabri S, Mohd Yahaya N, Mohd Shariff F, Mohamad Ali MS. Catalytically active inclusion bodies as a potential tool for biotechnology. Biotechnol Appl Biochem. 2024;71(6):1235–42. 38863240 10.1002/bab.2624

[52] Hrabárová E, Achbergerová L, Nahálka J. Insoluble protein applications: the use of bacterial inclusion bodies as biocatalysts. Methods Mol Biol. 2015;1258:411–22. 25447879 10.1007/978-1-4939-2205-5_24

[53] Georgiou G, Telford JN, Shuler ML, Wilson DB. Localization of inclusion bodies in *Escherichia coli* overproducing beta-lactamase or alkaline phosphatase. Appl Environ Microbiol. 1986;52(5):1157–61. 3539017 10.1128/aem.52.5.1157-1161.1986 PMC239190

[54] Flores SS, Nolan V, Perillo MA, Sánchez JM. Superactive β-galactosidase inclusion bodies. Colloids Surf B Biointerfaces. 2019;173:769–75. 30384274 10.1016/j.colsurfb.2018.10.049

[55] Han Y, Zhang X, Zheng L. Engineering actively magnetic crosslinked inclusion bodies of *Candida antarctica* lipase B: an efficient and stable biocatalyst for enzyme-catalyzed reactions. Mol Cata. 2021;504:111467.

[56] Kamel S, Walczak MC, Kaspar F, Westarp S, Neubauer P, Kurreck A. Thermostable adenosine 5’-monophosphate phosphorylase from *Thermococcus kodakarensis* forms catalytically active inclusion bodies. Sci Rep. 2021;11(1):16880. 34413335 10.1038/s41598-021-96073-5 PMC8376864

[57] Kloss R, Karmainski T, Jäger VD, Hahn D, Grünberger A, Baumgart M, Krauss U, Jaeger KE, Wiechert W, Pohl M. Tailor-made catalytically active inclusion bodies for different applications in biocatalysis. Cata Sci Technol. 2018;8(22):5816–26.

[58] Peternel S, Grdadolnik J, Gaberc-Porekar V, Komel R. Engineering inclusion bodies for Non denaturing extraction of functional proteins. Microb Cell Fact. 2008;1(7):34. 10.1186/1475-2859-7-34 PMC2630956 19046444

[59] Tokatlidis K, Dhurjati P, Millet J, Béguin P, Aubert J-P. High activity of inclusion bodies in *Escherichia coli* overproducing *Clostridium thermocellum* endoglucanase D. FEBS Lett. 1991;282(1):205–8. 2026260 10.1016/0014-5793(91)80478-l

[60] Singh A, Upadhyay V, Singh A, Panda AK. Structure-Function relationship of inclusion bodies of a multimeric protein. Front Microbiol. 2020;11:876. 32457730 10.3389/fmicb.2020.00876 PMC7225587

[61] Rinas U, Garcia-Fruitós E, Corchero JL, Vázquez E, Seras-Franzoso J, Villaverde A. Bacterial inclusion bodies: discovering their better half. Trends Biochem Sci. 2017;42(9):726–37. 28254353 10.1016/j.tibs.2017.01.005

[62] Slouka C, Kopp J, Hutwimmer S, Strahammer M, Strohmer D, Eitenberger E, Schwaighofer A, Herwig C. Custom made inclusion bodies: impact of classical process parameters and physiological parameters on inclusion body quality attributes. Microb Cell Fact. 2018;17(1):148. 30236107 10.1186/s12934-018-0997-5 PMC6148765

[63] Zhou B, Xing L, Wu W, Zhang XE, Lin Z. Small surfactant-like peptides can drive soluble proteins into active aggregates. Microb Cell Fact. 2012;11:2–9. 22251949 10.1186/1475-2859-11-10 PMC3398302

[64] Corchero JL, Viaplana E, Benito A, Villaverde A. The position of the heterologous domain can influence the solubility and proteolysis of beta-galactosidase fusion proteins in *E. coli*. J Biotechnol. 1996;48(3):191–200. 8861998 10.1016/0168-1656(96)01508-8

[65] Schrödel A, de Marco A. Characterization of the aggregates formed during Recombinant protein expression in bacteria. BMC Biochem. 2005;6:10. 15927061 10.1186/1471-2091-6-10 PMC1175841

[66] Mishra VK, Anantharamaiah GM, Segrest JP, Palgunachari MN, Chaddha M, Sham SW, Krishna NR. Association of a model class A (apolipoprotein) amphipathic alpha helical peptide with lipid: high resolution NMR studies of peptide.lipid discoidal complexes. J Biol Chem. 2006;281(10):6511–9. 16407255 10.1074/jbc.M511475200

[67] Naskar J, Palui G, Banerjee A. Tetrapeptide-based hydrogels: for encapsulation and slow release of an anticancer drug at physiological pH. J Phys Chem B. 2009;113(35):11787–92. 19708711 10.1021/jp904251j

[68] Salnikov ES, Anantharamaiah GM, Bechinger B. Supramolecular organization of Apolipoprotein-A-I-Derived peptides within Disc-like arrangements. Biophys J. 2018;115(3):467–77. 30054032 10.1016/j.bpj.2018.06.026 PMC6085177

[69] Zhang S, Holmes T, Lockshin C, Rich A. Spontaneous assembly of a self-complementary oligopeptide to form a stable macroscopic membrane. PNAS. 1993;90(8):3334–8. 7682699 10.1073/pnas.90.8.3334 PMC46294

[70] Lin Z, Zhou B, Wu W, Xing L, Zhao Q. Self-assembling amphipathic alpha-helical peptides induce the formation of active protein aggregates in vivo. Faraday Discuss. 2013;166:243–56. 24611280 10.1039/c3fd00068k

[71] Quax TE, Claassens NJ, Söll D, van der Oost J. Codon bias as a means to Fine-Tune gene expression. Mol Cell. 2015;59(2):149–61. 26186290 10.1016/j.molcel.2015.05.035 PMC4794256

[72] Restrepo-Pineda S, Sánchez-Puig N, Pérez NO, García-Hernández E, Valdez-Cruz NA, Trujillo-Roldán MA. The pre-induction temperature affects Recombinant HuGM-CSF aggregation in thermoinducible *Escherichia coli*. Appl Microbiol Biotechnol. 2022;106(8):2883–902. 35412129 10.1007/s00253-022-11908-z PMC9002048

[73] Valdez-Cruz NA, Reynoso-Cereceda GI, Pérez-Rodriguez S, Restrepo-Pineda S, González-Santana J, Olvera A, Zavala G, Alagón A, Trujillo-Roldán MA. Production of a Recombinant phospholipase A2 in *Escherichia coli* using resonant acoustic mixing that improves oxygen transfer in shake flasks. Microb Cell Fact. 2017;16(1):129. 28743267 10.1186/s12934-017-0746-1 PMC5526256

[74] Zandomeneghi G, Krebs MR, McCammon MG, Fändrich M. FTIR reveals structural differences between native beta-sheet proteins and amyloid fibrils. Protein Sci. 2004;13(12):3314–21. 15537750 10.1110/ps.041024904 PMC2287307

[75] Natalello A, Doglia SM. Insoluble protein assemblies characterized by fourier transform infrared spectroscopy. In: García-Fruitós E, editor. Insoluble proteins. Methods in molecular biology (methods and protocols). New York NY: Humana; 2015. pp. 347–69. 10.1007/978-1-4939-2205-5_20 25447875

[76] Ami D, Natalello A, Taylor G, Tonon G. Doglia: structural analysis ofprotein inclusion bodies by fourier transform infrared microspectroscopy,Biochim. Biophys Acta. 2006;1764(4):793–9. 10.1016/j.bbapap.2005.12.005 16434245

[77] Harris JR, Scheffler D. Routine Preparation of air-dried negatively stained and unstained specimens on Holey carbon support films: a review of applications. Micron. 2002;33(5):461–80. 11976034 10.1016/s0968-4328(01)00039-7

[78] Margot J, Bennati-Granier C, Maillard J, Blánquez P, Barry DA, Holliger C. Bacterial versus fungal laccase: potential for micropollutant degradation. AMB Express. 2013;3(1):63. 24152339 10.1186/2191-0855-3-63 PMC3819643

[79] Neelkant KS, Shankar K, Jayalakshmi SK, Sreeramulu K. Purification, biochemical characterization, and facile immobilization of laccase from *Sphingobacterium ksn-11* and its application in transformation of diclofenac. Appl Biochem Biotechnol. 2020;192(3):831–44. 32601857 10.1007/s12010-020-03371-1

[80] Tonin F, Melis R, Cordes A, Sanchez-Amat A, Pollegioni L, Rosini E. Comparison of different microbial laccases as tools for industrial uses. N Biotechnol. 2016;33(3):387–98. 26844639 10.1016/j.nbt.2016.01.007

[81] Ouyang J, Zhao Z, Suib SL, Yang H. Degradation of congo red dye by a Fe2O3@CeO2-ZrO2/Palygorskite composite catalyst: synergetic effects of Fe2O3. J Colloid Interface Sci. 2019;539:135–45. 30579217 10.1016/j.jcis.2018.12.052

[82] Yaseen DA, Scholz M. Textile dye wastewater characteristics and constituents of synthetic effluents: a critical review. Int J Environ Sci Technol. 2019;16:1193–226.

[83] Steinegger M, Söding J. MMseqs2 enables sensitive protein sequence searching for the analysis of massive data sets. Nat Biotechnol. 2017;35(11):1026–8. 29035372 10.1038/nbt.3988

[84] Wohlwend J, Corso G, Passaro S, Getz N, Reveiz M, Leidal K, Swiderski W, Atkinson L, Portnoi T, Chinn I, Silterra J, Jaakkola T, Barzilay R. Boltz-1 democratizing biomolecular interaction modeling. BioRxiv [Preprint]. 2025;2024(1119624167). 10.1101/2024.11.19.624167.

[85] Kelley LA, Gardner SP, Sutcliffe MJ. An automated approach for clustering an ensemble of NMR-derived protein structures into conformationally related subfamilies. Protein Eng. 1996;9(11):1063–5. 8961360 10.1093/protein/9.11.1063

[86] He W, Yiru, Wang, Ge X, Li Y. Activity and stability improvement: structure-function insights into CotA from *Bacillus subtilis*. Int J Biol Macromol. 2025;318(Pt 4):145156. Erratum in: Int J Biol Macromol. 2025;319(Pt 2):145952. 10.1016/j.ijbiomac.2025.145156 40505904

[87] Mohajeri A, Pilehvar-Soltanahmadi Y, Pourhassan-Moghaddam M, Abdolalizadeh J, Karimi P, Zarghami N. Cloning and expression of Recombinant human endostatin in periplasm of *Escherichia coli* expression system. Adv Pharm Bull. 2016;6(2):187–94. 27478780 10.15171/apb.2016.026 PMC4961976

[88] Menzella HG. Comparison of two codon optimization strategies to enhance Recombinant protein production in *Escherichia coli*. Microb Cell Fact. 2011;10:15. 21371320 10.1186/1475-2859-10-15 PMC3056764

[89] Wu W, Xing L, Zhou B, Lin Z. Active protein aggregates induced by terminally attached self-assembling peptide ELK16 in *Escherichia coli*. Microb Cell Fact. 2011;10:9. 21320350 10.1186/1475-2859-10-9 PMC3045283

[90] Brauneck G, Engel D, Grebe LA, Hoffmann M, Lichtenberg PG, Neuß A, Mann M, Magnus JB. Pitfalls in early bioprocess development using shake flask cultivations. Eng Life Sci. 2025;25(1):e70001. 39877379 10.1002/elsc.70001 PMC11773345

[91] Restrepo-Pineda S, Bando-Campos CG, Valdez-Cruz NA, Trujillo-Roldán MA. Recombinant production of ESAT-6 antigen in thermoinducible *Escherichia coli*: the role of culture scale and temperature on metabolic response, expression of chaperones, and architecture of inclusion bodies. Cell Stress Chaperones. 2019;24(4):777–92. 31165436 10.1007/s12192-019-01006-x PMC6629757

[92] Reynoso-Cereceda GI, Valdez-Cruz NA, Pérez NO, Trujillo-Roldán MA. A comprehensive study of glucose and oxygen gradients in a scaled-down model of Recombinant HuGM-CSF production in thermoinduced *Escherichia coli* fed-batch cultures. Prep Biochem Biotechnol. 2024;54(10):1263–74. 38701182 10.1080/10826068.2024.2347403

[93] Mohammadian M, Fathi-Roudsari M, Mollania N, Badoei-Dalfard A, Khajeh K. Enhanced expression of a Recombinant bacterial laccase at low temperature and microaerobic conditions: purification and biochemical characterization. J Ind Microbiol Biotechnol. 2010;37(8):863–9. 20473548 10.1007/s10295-010-0734-5

[94] Krauss U, Jäger VD, Diener M, Pohl M, Jaeger KE. Catalytically-active inclusion bodies-Carrier-free protein immobilizates for application in biotechnology and biomedicine. J Biotechnol. 2017;258:136–47. 28465211 10.1016/j.jbiotec.2017.04.033

[95] Upadhyay AK, Murmu A, Singh A, Panda AK. Kinetics of inclusion body formation and its correlation with the characteristics of protein aggregates in *Escherichia coli*. PLoS ONE. 2012;7(3):e33951. 22479486 10.1371/journal.pone.0033951 PMC3315509

[96] Baneyx F, Mujacic M. Recombinant protein folding and misfolding in *Escherichia coli*. Nat Biotechnol. 2004;22(11):1399–408. 15529165 10.1038/nbt1029

[97] de Marco A, Ferrer-Miralles N, Garcia-Fruitós E, Mitraki A, Peternel S, Rinas U, Trujillo-Roldán MA, Valdez-Cruz NA, Vázquez E, Villaverde A. Bacterial inclusion bodies are industrially exploitable amyloids. FEMS Microbiol Rev. 2019;43(1):53–72. 30357330 10.1093/femsre/fuy038

[98] Peternel S, Komel R. Active protein aggregates produced in *Escherichia coli*. Int J Mol Sci. 2011;12(11):8275–87. 22174663 10.3390/ijms12118275 PMC3233469

[99] Samak N, Tan Y, Sui K, Ting-Ting X, Wang K, Guo C, Liu C. CotA laccase immobilized on functionalized magnetic graphene oxide nano-sheets for efficient biocatalysis. Mol Catal. 2017;445:269–78.

[100] Bahamondes C, Illanes A, Pouchucq L. Effect of external diffusional restrictions in immobilized enzymes in stirred reactors. Biocatal Biotransform. 2025;43(3):28–244.

[101] Müller J, Pfleiderer G. Factors affecting the activity of immobilized enzymes, I. Diffusional limitation. Hoppe Seylers Z Physiol Chem. 1980;361(5):675–80. 7429423 10.1515/bchm2.1980.361.1.675

[102] Mirdita M, Schütze K, Moriwaki Y, Heo L, Ovchinnikov S, Steinegger M. ColabFold: making protein folding accessible to all. Nat Methods. 2022;19(6):679–82. 35637307 10.1038/s41592-022-01488-1 PMC9184281

[103] Baek M, DiMaio F, Anishchenko I, Dauparas J, Ovchinnikov S, Lee GR, Wang J, Cong Q, Kinch LN, Schaeffer RD, Millán C, Park H, Adams C, Glassman CR, DeGiovanni A, Pereira JH, Rodrigues AV, van Dijk AA, Ebrecht AC, Opperman DJ, Sagmeister T, Buhlheller C, Pavkov-Keller T, Rathinaswamy MK, Dalwadi U, Yip CK, Burke JE, Garcia KC, Grishin NV, Adams PD, Read RJ, Baker D. Accurate prediction of protein structures and interactions using a three-track neural network. Science. 2021;373(6557):871–6. 34282049 10.1126/science.abj8754 PMC7612213

[104] Zheng W, Zhang C, Li Y, Pearce R, Bell EW, Zhang Y. Folding non-homologous proteins by coupling deep-learning contact maps with I-TASSER assembly simulations. Cell Rep Methods. 2021;1(3):100014. 34355210 10.1016/j.crmeth.2021.100014 PMC8336924

[105] Żur J, Wojcieszyńska D, Guzik U. Metabolic responses of bacterial cells to immobilization. Molecules. 2016;21(7):958. 27455220 10.3390/molecules21070958 PMC6273605

[106] Martins SCS, Martins CM, Fiuza LMCG, Santaella ST. Immobilization of microbial cells: A promising tool for treatment of toxic pollutants in industrial wastewater. Afr J Biotechnol. 2013:124412–8.

[107] Dong CD, Tiwari A, Anisha GS, Chen CW, Singh A, Haldar D, Patel AK, Singhania RR. Laccase: A potential biocatalyst for pollutant degradation. Environ Pollut. 2023;319:120999. 36608728 10.1016/j.envpol.2023.120999

[108] Ma X, Liu L, Li Q, Liu Y, Yi L, Ma L, Zhai C. High-level expression of a bacterial laccase, CueO from *Escherichia coli* K12 in *Pichia pastoris* GS115 and its application on the decolorization of synthetic dyes. Enzyme Microb Technol. 2017;103:34–41. 28554383 10.1016/j.enzmictec.2017.04.004

[109] Datta S, Christena LR, Rajaram YR. Enzyme immobilization: an overview on techniques and support materials. 3 Biotech. 2013;3(1):1–9. 28324347 10.1007/s13205-012-0071-7 PMC3563746

[110] El-Bendary MA, Ezzat SM, Ewais EA, Al-Zalama MA. Efficient immobilization of highly stable *Bacillus amyloliquefaciens* spore laccase for biodecolorization of textile dyes in water. Environ Sci Eur. 2024;36:36.

[111] Fernández-Fernández M, Sanromán MÁ, Moldes D. Recent developments and applications of immobilized laccase. Biotechnol Adv. 2013;31(8):1808–25. 22398306 10.1016/j.biotechadv.2012.02.013

[112] Hong J, Jung D, Park S, Oh Y, Oh KK, Lee SH. Immobilization of laccase via cross-linked enzyme aggregates prepared using Genipin as a natural cross-linker. Int J Biol Macromol. 2021;169:541–50. 33358952 10.1016/j.ijbiomac.2020.12.136

[113] Ölçücü G, Jaeger KE, Krauss U. Design, Production, and characterization of catalytically active inclusion bodies. Methods Mol Biol. 2023;2617:49–74. 36656516 10.1007/978-1-0716-2930-7_4

[114] Younus H, Khan MA, Khan A, Alhumaydhi FA. Eco-Friendly biocatalysts: laccase Applications, Innovations, and future directions in environmental remediation. Catalysts. 2025;15(10):921.

[115] Ren D, Wang Z, Jiang S, Yu H, Zhang S, Zhang X. Recent environmental applications of and development prospects for immobilized laccase: a review. Biotechnol Gen Eng Rev. 2020;36(2):81–131. 10.1080/02648725.2020.1864187 33435852

[116] Girelli AM, Scuto FR. Spent grain as a sustainable and low-cost carrier for laccase immobilization. Waste Manag. 2021;128:114–21. 33984682 10.1016/j.wasman.2021.04.055

[117] Sharma R, Anupa A, Rathore AS. Refolding of proteins expressed as inclusion bodies in *E. coli*. Methods Mol Biol. 2023;2617:201–08. 36656526 10.1007/978-1-0716-2930-7_14

[118] Hojnik Podrepšek G, Knez Ž, Leitgeb M. The synthesis of (Magnetic) crosslinked enzyme aggregates with Laccase, Cellulase, β-Galactosidase, and transglutaminase. Front Bioeng Biotechnol. 2022;10:813919. 35309987 10.3389/fbioe.2022.813919 PMC8927696

[119] Xu R, Zhang X, Zelekew OA, Schott E, Wu YN. Improved stability and activity of laccase through de Novo and post-synthesis immobilization on a hierarchically porous metal-organic framework (ZIF-8). RSC Adv. 2023;13(25):17194–201. 37304779 10.1039/d3ra01571h PMC10248541

[120] Iafolla MA, Mazumder M, Sardana V, Velauthapillai T, Pannu K, McMillen DR. Dark proteins: effect of inclusion body formation on quantification of protein expression. Proteins. 2008;72(4):1233–42. 18350571 10.1002/prot.22024

[121] Mairhofer J, Scharl T, Marisch K, Cserjan-Puschmann M, Striedner G. Comparative transcription profiling and in-depth characterization of plasmid-based and plasmid-free *Escherichia coli* expression systems under production conditions. Appl Environ Microbiol. 2013;79(12):3802–12. 23584782 10.1128/AEM.00365-13 PMC3675926

[122] Liu J, Chen J, Zuo K, Li H, Peng F, Ran Q, Wang R, Jiang Z, Song H. Chemically induced oxidative stress improved bacterial laccase-mediated degradation and detoxification of the synthetic dyes. Ecotoxicol Environ Saf. 2021;15(226):112823. 10.1016/j.ecoenv.2021.112823 34597843

[123] Abioye OP, Umaru S, Aransiola SA, Oyewole OA, Maddela NR, Prasad R. Production of laccase by *Bacillus subtilis* and *Aspergillus Niger* for treatment of textile effluent. Sustainable Chem Environ. 2025;9:100222.

