# Supplementary figures and images for: Catalytically active inclusion bodies of Bacillus clausii laccase protein recombinantly produced in E. coli for dye decolorization
Source: Microb Cell Fact. 2026 Mar 6;25:95. doi: 10.1186/s12934-026-02949-4 (PMC13081310; doi:10.1186/s12934-026-02949-4)

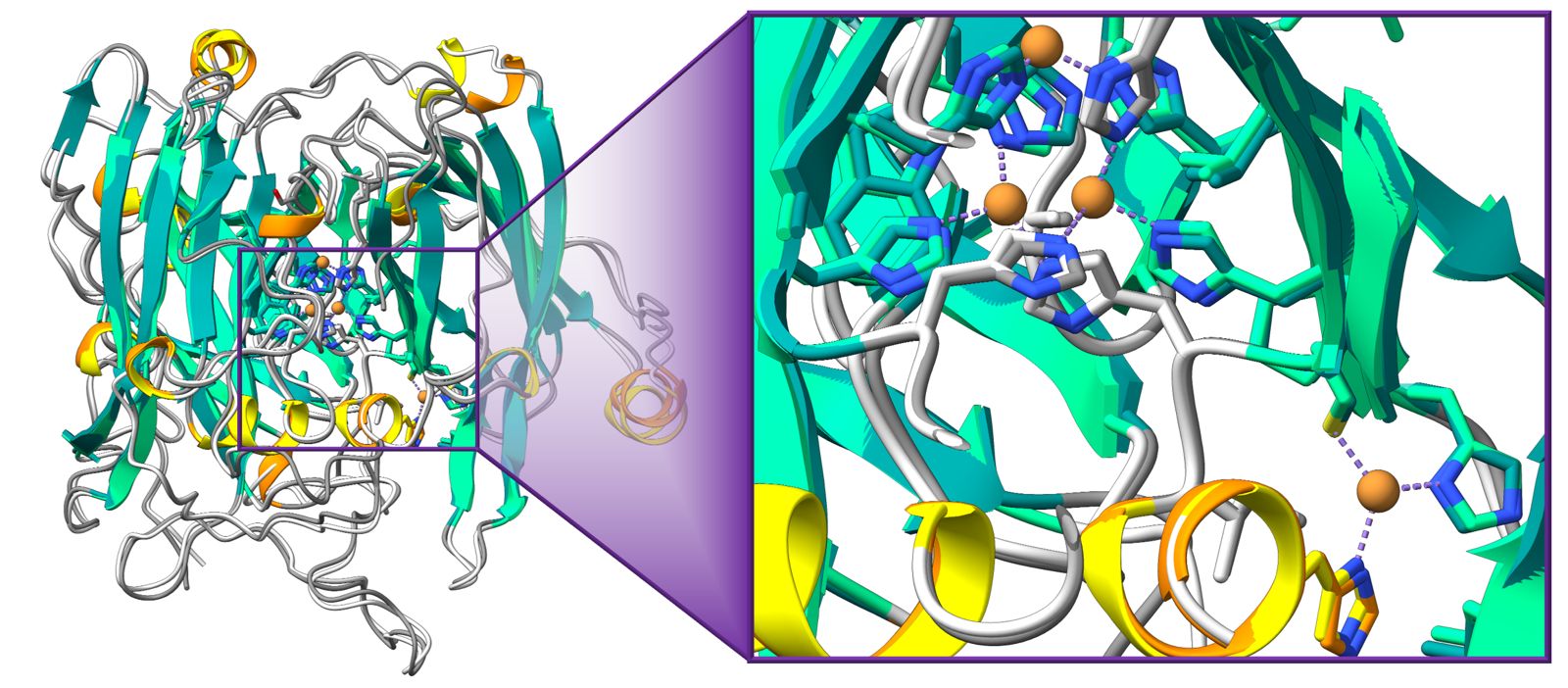

Supplement: Supplementary file 1 — Supplementary Material 1. [file 12934_2026_2949_MOESM1_ESM.jpeg]
